# Shared Genetic Basis, Biological Function and Causal Relationship Between Sleep Traits and Hypothyroidism: Evidence from a Comprehensive Genetic Analysis

**DOI:** 10.2174/0118715303348704250227014802

**Published:** 2025-04-03

**Authors:** Yinli Shi, Ming Guo, Yuedan Wang, Guoduan Zeng, Wenting Li, Mianhua Wu, Bo Li

**Affiliations:** 1 Institute Of Basic Research in Clinical Medicine, China Academy of Chinese Medical Sciences, Beijing, 100700, China;; 2 Zhongda Hospital Southeast University, Southeast University, Nanjing, 210023, China;; 3 Institute of Digestive Diseases, Xiyuan Hospital of China Academy of Chinese Medical Sciences, Beijing, 100091, China;; 4 Jinjiang Hospital of Traditional Chinese Medicine, Fujian University of Chinese Medicine, Fujian, 362211, China;; 5 The First Clinical Medical College, Nanjing University of Chinese Medicine, Nanjing, 210023, China

**Keywords:** Sleep traits, hypothyroidism, mendelian randomization study, LDSC, CPASSOC, genetic similarities

## Abstract

**Background:**

This research attempts to clarify whether there are any genetic similarities between sleep traits and hypothyroidism based on publicly accessible large-scale genome-wide association studies.

**Methods:**

The methodology included colocalization analysis, cross-phenotype association analysis, and linkage disequilibrium score regression analysis to find common genetic overlap. Through tissue function specificity and functional mapping, we were able to identify the shared genetic level. Genetic instrumental factors were used for causal inference in two-sample univariate and multivariable Mendelian randomization analyses.

**Results:**

A hereditary correlation between hypothyroidism and napping during the day and getting up in the morning (r_g_= -0.0982, *P*= 0.0007; r_g_= -0.101, *P*= 0.0001). MAGI3, and HLA-DRB1 BX296568.1 may be potential targets for shared treatments. Colocalization and tissue-specific analysis demonstrated that the common genes and SNPs were identified in the thyroid, lung, brain, and lymphatic tissues. Functional analysis emphasized the importance of these common genes in processes like as protein transport, inflammatory response, and MHC class II protein synthesis. Furthermore, an association has been established between hypothyroidism and sleep duration (IVW, OR 1.5208; 95% CI 1.1142-2.0758, *P*=0.0082) and getting up in the morning (IVW, OR 1.8375; 95%CI: 1.4502-2.3284, *P*=4.73E-07). Furthermore, the reverse MR analysis revealed no causal connection between aberrant sleep traits and hypothyroidism. The enduring impact of insomnia on hypothyroidism persists despite controlling for alcohol consumption and smoking habits.

**Conclusion:**

Certain genetic correlations between sleep traits and hypothyroidism have been emphasized. These findings may elucidate the origin of comorbidity and have implications for future clinical trials.

## 1 INTRODUCTION

Hypothyroidism is a systemic hypometabolic condition caused by reduced production of thyroid hormones due to thyroid injury, autoimmune thyroiditis, and abnormal iodine levels. Overt hypothyroidism and subclinical hypothyroidism represent the two primary manifestations of hypothyroidism [1, 2]. Studies have shown that the prevalence of overt and subclinical hypothyroidism is 3.8% and 0.37%, respectively, with higher incidence rates observed in older persons and women [3]. The manifestations of hypothyroidism generally evolve gradually for the disease. Common yet often non-specific symptoms include constipation, fatigue, and a feeling of cold intolerance [4, 5].

A variety of attributes, including duration, consistency, satisfaction, alertness, and efficiency, can be employed to assess the efficacy of sleep. Sleep disturbance is a significant and prevalent public health issue impacting millions of individuals annually, with varying degrees of adverse consequences on the immune system [6, 7]. The potential association between hypothyroidism and sleep disturbances appears to be underexplored. Despite the lack of a strong physiological or experimental link between hypothyroidism and sleep disturbances, it is unclear whether sleep disturbances directly cause hypothyroidism or if they are merely a risk factor for its development [8]. Observational studies have linked untreated subclinical hypothyroidism with diminished sleep duration, inferior quality, and prolonged sleep latency; additional risk variables encompass smoking, alcohol consumption, and a lower body mass index [9, 10]. Furthermore, research indicates that individuals with hypothyroidism experience reduced sleep latency, a lower incidence of late-night sleeping during weekdays, and diminished total and subjective sleep quality. Irrespective of thyroid peroxidase antibody (TPOAb) levels, these people exhibit statistically significant reductions in general health-related quality of life and sleep quality [11, 12]. Additionally, another study suggests that variations in sleep duration, whether extended or reduced, may disrupt circadian rhythms, thereby impacting thyroid function and increasing the risk of hypothyroidism [13]. Moreover, sleep is a multifaceted notion encompassing chronotype, morning awakening, sleep duration, nap during the day, and insomnia. Establishing a causal association between sleep disruptions and hypothyroidism is challenging due to confounding variables and the extended follow-up durations necessary for observational studies. It is essential to investigate potential genetic correlations to establish a causal relationship between hypothyroidism and other aberrant sleep habits [14]. An effective approach is required to prove that sleep traits are causally related to negative consequences in various situations.

Numerous computational techniques have recently arisen to address these difficulties. Genome-wide association studies (GWAS), including genetic correlation analysis, cross-trait meta-analysis, and Mendelian randomization (MR), provide extensive insights into genetic correlation and shared loci from numerous perspectives [15, 16]. These methods validate causal links between diseases, showcasing their dependability as analytical tools that utilize extensive GWAS data while circumventing the restrictions inherent in observational investigations [17, 18]. The limitations of observational studies might be addressed by large-scale data analysis from GWAS. Although limited research has been conducted on hypothyroidism using these techniques, they have been extensively applied to investigate the causal relationships between sleep and various diseases, including cancer, gastrointestinal disorders, rheumatic conditions, and cardiovascular diseases [19, 20]. By evaluating the association between different sleep traits and hypothyroidism, this study aims to elucidate the potential causal relationship between sleep traits and hypothyroidism, thereby providing novel therapeutic approaches.

## 2 MATERIALS AND METHODS

### 2.1 Data Source

The data for the two-sample MR analysis was sourced from publicly accessible GWAS databases. Data on sleep traits, encompassing insomnia, sleep duration, nap during the day, getting up in the morning, chronotype, and snoring, were obtained from the IEU Open GWAS database (https://gwas.mrcieu.ac.uk/). The sleep duration was determined by asking the UK Biobank the following question: “How many hours do you sleep in a day?” Short sleepers (less than 7 hours per day), normal sleepers (between 7 and 9 hours per day—serving as the benchmark for comparing short and long sleepers), and long sleepers (more than 9 hours per day) were the categories used to classify sleep duration [21]. In “Do you nap during the day?” the question assessed napping during the day and had one of four possible answers [22, 23]: “never/rarely,” “sometimes,” “usually,” and “don't want to answer.” The significant self-report was not too difficult to find, and getting up in the morning was assessed based on its ease of location. “Do you consider yourself to be (1) a “morning” person, (2) more morning than evening, (3) more evening than morning, or (4) an “evening” person?” was the question utilized for gathering self-report information regarding chronotype. The snoring complaints from a partner, relative, or friend led to the hypothesis of a genetic link to non-snoring.

Furthermore, aggregated GWAS data for weekly alcohol consumption and smoking initiation were acquired from the same database. Data about hypothyroidism were obtained from the FinnGen database (https://www.finngen.fi/fi), with comprehensive details shown in Table **1** [24]. Data regarding ethical approval and authorization for the aggregate statistics was obtained from the original publication, and all datasets included in this study were sourced from the public domain.

### 2.2 Genetic Correlation Analysis

LDSC v1.0.1 (https://github.com/bulik/ldsc) is used to evaluate the relationship between sleep characteristics and hypothyroidism, including liability-scale heritability (h2) and genetic correlation (rg). In linkage disequilibrium score regression (LDSC), the linkage disequilibrium (LD) score of the Single nucleotide polymorphism (SNP) site serves as the dependent variable, whereas this value functions as the independent variable of the approach. A self-defined statistic aligns with the chi-square distribution. To ascertain whether any confounding variables influence the outcomes of the GWAS analysis, a linear regression has been used to model the relationship between the LD score and the chi-square statistic. It can be inferred that confounding factors alone do not account for the overall phenotypic correlation if the genetic association is both statistically and quantitatively significant [25, 26].

### 2.3 Cross-Trait Meta-Analysis

The Cross-Phenotype Association Analysis (CPASSOC) approach was utilized to evaluate effect estimation and standard error in GWAS precision measures [27]. This research utilized the installation packages “dplyr” and “data.table.” The goal of the approach was to identify shared genetic variations between SNPs and both traits. Paired Sample Heterogeneity (SHet) was utilized to differentiate and consolidate data related to metabolic syndrome and its components across diverse rheumatic conditions. Relevant SNPs were identified using the following PLINK function parameters: clump-p1 5e-8, clump-p2 1e-5, clump-r2 0.2, clump 500kb. SNPs were deemed significant if the P-value for a single characteristic was less than 1E-05 and the CPASSOC value was below 5E-08 [28, 29]. Thereafter, SNPs discovered with CPASSOC were functionally annotated, and gene mapping was performed utilizing the Ensembl Variant Effect Predictor (VEP) and the Genotype Tissue Expression Project (GTEx) database [30].

### 2.4 Coloc Analysis

The CPASSOC led to the integration of genetic loci associated with specific behaviors. The colocalization approach (COLOC) was employed to ascertain whether the similar genetic variation in the loci is responsible for both traits [31]. Posterior probabilities for five unique hypotheses regarding the sharing of causal changes within a genomic area are calculated using a Bayesian technique. H0 (no association), H1 or H2 (connection with a specific characteristic), H3 (association with both traits, involving two separate SNPs), and H4 (association with both traits, involving one shared SNP) represent several of these hypotheses. A locus is considered colocalized if the PPH4 value exceeds 0.7 [32]. The “coloc” package facilitated this analysis.

### 2.5 Functional Enrichment Analysis

Cytoscape 3.6.1 was utilized to visualize the resultant protein-protein interaction (PPI) target networks for these connections. Before commencing the analysis, please pre- download the following packages: “dplyr,” “org.Hs.eg.db,” “enrichplot,” “ggplot2,” “clusterProfiler,” and “DOSE.” Establish *P*<0.05 and *Q*<0.05 as the threshold parameters [33]. Subsequently, investigate the common genes utilizing the Kyoto Encyclopedia of Genes and Genomes (KEGG) pathway enrichment analysis and gene ontology (GO) functional analysis.

### 2.6 MR Analysis

#### 2.6.1 MR Hypothesis and Analysis of SNP Data

The forward Mendelian Randomization hypothesis and analysis of SNP data related to hypothyroidism were identified as a result of the two-sample Mendelian Randomization study, using sleep traits as exposure variables. The reverse Mendelian randomization hypothesis modifies the exposure and outcome variables. To identify SNPs of significance according to the *P* < 5E-08 criterion, obtain the “TwoSampleMR” package [34, 35]. The LD of pertinent SNPs was evaluated according to the following criteria: r^2^≤0.001; clumping window, 10,000 kb.

#### 2.6.2 Two-Sample Univariate and Nultivariate MR Analysis

The study included various methodologies to evaluate the causal relationship regarding the consequences of hypothyroidism. The inverse-variance weighted (IVW) method was the primary statistical method employed. When all genetic variations fit the assumptions of prior instrumental variables, the IVW method consistently produced an assessment of the causal impact of exposure factors on outcomes [36]. Additionally, a reliable causal effect assessment of many genetic variants using aggregated GWAS data was achieved under minimal assumptions utilizing the MR-Egger and weighted median methodologies, which rely on assumptions on instrumental strength that are independent of direct effects [37, 38]. Funnel plots were generated, and intercepts represent estimates of the average extent of pleiotropic effects of genetic variation. The slope of the linear regression indicates the estimates of the causal effects.

Prior observational studies have identified significant risk factors for the onset of hypothyroidism, including smoking and alcohol consumption [39]. A multivariate Mendelian randomization (MVMR) study was conducted utilizing the “Mendelian randomization” package to assess the direct causal impact of alcohol and smoking on the risk of rheumatic diseases [40, 41].

#### 2.6.3 Sensitivity Analysis

The study evaluated the heterogeneity between each SNP estimate using Cochran's Q statistic. A random effects model was applied to the identification of heterogeneity (Cochrane's Q *p* < 0.05). The MR-PRESSO methodology was utilized to conduct outlier and distortion analyses to identify and exclude outliers that could compromise the results from further examination [42]. If *P* > 0.05, there is a minimal likelihood of pleiotropy in the causal analysis, and its effect is deemed insignificant [43]. The inquiry also examined the potential confounding variables in the horizontal pleiotropy assessments. The stability of the impact size was evaluated using the leave-one-out method, which was also employed to identify specific SNPs that contributed much more to the association than others.

The study utilized R (version 4.1.3) for all statistical calculations. A double-tailed *P* < 0.05 indicated statistical significance. Fig. (**1a**) illustrates the exact operational processes of this study, while Fig. (**1b**) outlines the specific procedures of the MR hypothesis.

## 3 RESULTS

### 3.1 Genetic Association Between Multiple Sleep Traits and Hypothyroidism

The analysis revealed that the maximum overlap rate between sleep parameters and hypothyroidism samples was below 10%. The exposure and outcome data were obtained from the Finnish database and the UKB database, respectively, demonstrating a distinct absence of sample overlap.

Using LDSC, the heritability h2 values of hypothyroidism and sleep traits such as sleep duration, napping during the day, getting up in the morning, insomnia, chronotype, snoring were evaluated, with results of 0.1228 (SE=0.0075), 0.0592 (SE=0.0069), 0.0597 (SE=0.0023), 0.0695 (SE=0.0026), 0.0659 (SE=0.0027), 0.1228 (SE=0.0075), and 0.0786 (SE=0.0032), respectively. Table **2** reveals a genetic association between hypothyroidism and sleep duration and getting up in the morning (r_g_= -0.0982, *P*= 0.0007; r_g_= -0.101, *P*= 0.0001).

### 3.2 Shared Loci Between Sleep Traits and Hypothyroidism

We discovered a total of 15 index loci and 23 mapped genes common to sleep characteristics and hypothyroidism. Insomnia and hypothyroidism simultaneously exhibit 4 index loci, comprising a total of 9 genes (SNP: rs1230666, rs28533694, rs9270493, rs9273363, mapped genes: MAGI3, HLA-DRB1, BX296568.1, HLA-DQB1, HLA-DQA1, AL731683.1, BX927168.3, BX927168.1, AL935026.1). Sleep duration, nap during the day, getting up in the morning day, and getting up in the morning are associated with loci at positions 9, 6, and 6 on the index, which correspond to mapped genes at positions 10, 13, and 14. In addition, chronotype and snoring display shared index loci of 6 and 3, correspondingly mapping to 10 and 5 genes. The most notable SNP for sleep traits and hypothyroidism is rs1230666 (*PCPASSOC*=1.23E-11, mapped gene: MAGI3). Fig. (**2**) and Table **3** provide more information.

Subsequent colocalization analysis revealed seven loci with a PPH4 value beyond 70% (Table **4**). The relevant SNPs, rs1049225, rs9273330, rs6472, and rs9270911, were common to both sleep traits and hypothyroidism, representing the most significant locus (PPH4=0.999). The loci were located at chr: bp 6:32627747, 6:32623300, 6:32007849, and 6:32572202. It mapped to four genes (HLA-DRB1, HLA-DQA1, SKIV2L, and PRRC2A).

### 3.3 Analysis of Tissue Function Specificity Based on Shared Genes

**Fig. (3):**
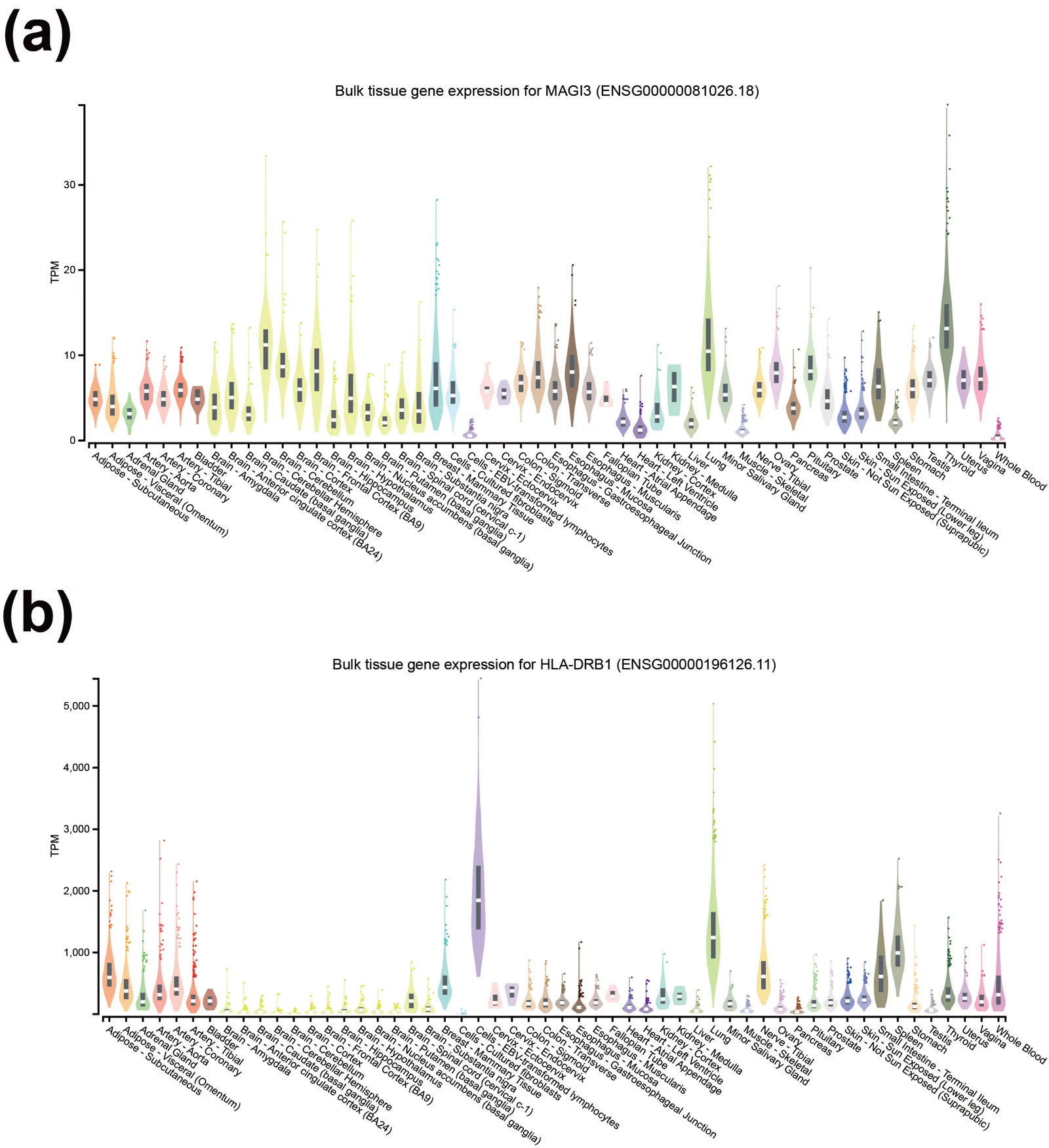
The result of tissue enrichment in GTEx of shared genes. (**a**) MAGI3. (**b**) HLA-DRB1.

The Venn diagram of the six sleep traits identified three overlapping shared genes (MAGI3, BX296568.1, HLA-DRB1) and a shared locus rs1230666. The pituitary gland, thyroid, lung, and brain exhibited substantial amounts of the shared gene MAGI3, whereas the thyroid, lung, lymphatic system, and arteries displayed elevated expression of the shared gene HLA-DRB1. However, there is no information available concerning the tissue expression of the BX296568.1 gene (Fig. **3**).

### 3.4 Biological Mechanisms Shared Between Sleep Traits and Hypothyroidism

**Fig. (4):**
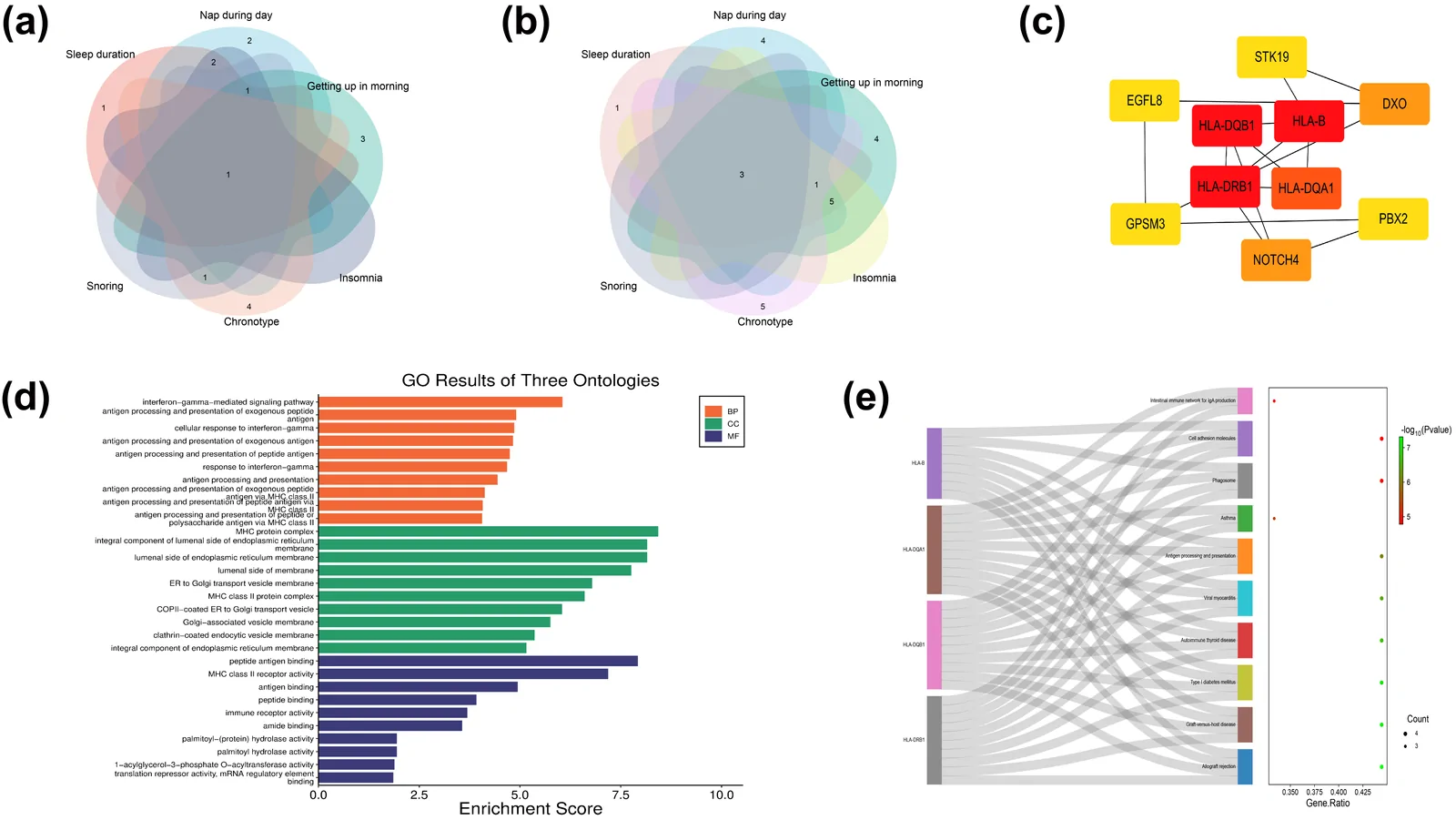
(**a**) The venn diagram reflects the intersection of SNPs for sleep traits. (**b**) The intersection of mapped genes in sleep traits. (**c**) The PPI network (MCC Top10). (**d**) GO functional analysis of a variety of shared genes (Top 10). (**e**) KEGG analysis of a variety of shared genes (Top 10).

The outcomes of the GO enrichment analysis, comprising 139 biological process (BP), 30 cellular components (CC), and 24 molecular functions (MF) items, indicated that the mapped genes were significantly associated with antigen processing and presentation of exogenous antigens, MHC class II protein production and transport, as well as antigen and peptide binding. Genes associated with many disorders exhibited enrichment in multiple immune signaling pathways, such as autoimmune thyroid disease, Th1 and Th2 cell differentiation, and Th17 cell differentiation. Moreover, these genes have been associated with disorders including cancer, rheumatic immunological disease, and infectious diseases, among others (Fig. **4**).

### 3.5 MR Analysis

#### 3.5.1 Insomnia and Hypothyroidism

**Fig. (5):**
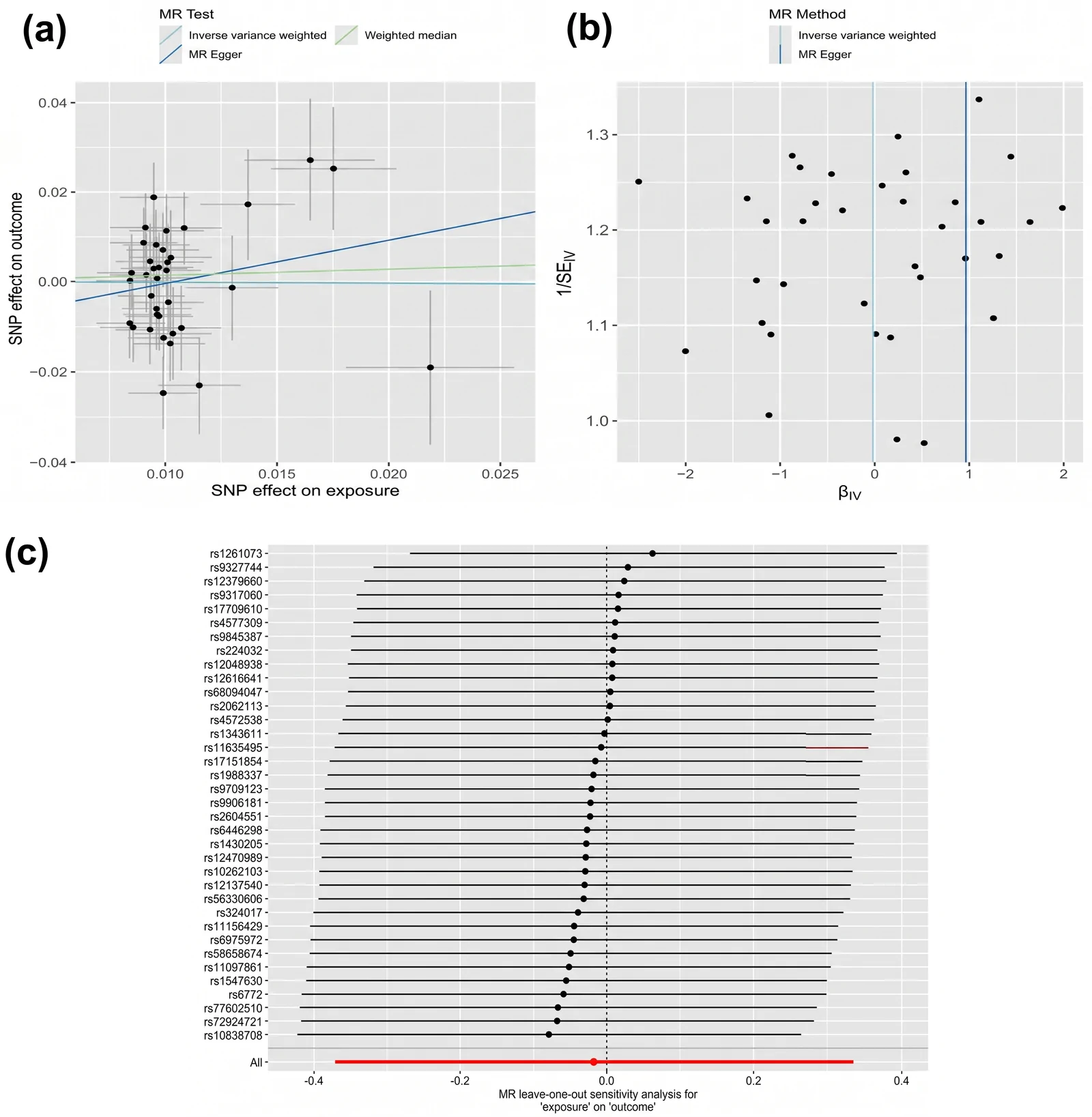
The causal effect of insomnia on hypothyroidism risk. (**a**) Scatter plot. (**b**) Funnel plot. (**c**) Leave-one-out plots.

Following the exclusion of 1,925 SNPs linked to insomnia, 42 SNPs persisted. Consequently, one SNP not associated with hypothyroidism and five palindromic SNPs were omitted, resulting in 36 SNPs for the MR analysis. The IVW analysis indicated no significant correlation between hypothyroidism and insomnia (IVW, OR 0.9820; 95% CI 0.6901-1.3973; *P*=0.9198). The MR-Egger and weighted median approaches revealed no significant difference between the intercept and zero (*P*=0.3724 and *P*=0.4994). The MR-PRESSO approach revealed rs1261073 as an outlier; nevertheless, its removal resulted in a non-significant heterogeneity (*Q*=47.06, *P*=0.0673), and the MR analysis indicated no substantial alteration (IVW, OR 1.0636; 95% CI 0.7645-1.4797; *P*=0.7144). The MR-Egger regression indicated the absence of horizontal pleiotropy (*P*=0.3677). The data were substantial and devoid of weak instrument bias (F=37.54). The leave-one-out study verified that the beta value was above zero, signifying insensitivity to genetic variation. After adjusting for alcohol intake and smoking, MVMR analysis revealed no significant association between insomnia and hypothyroidism. (IVW OR 1.1007; 95% CI: 0.6842–1.7708, *P*=0.6924) (Fig. **5**).

#### 3.5.2 Sleep Duration and Hypothyroidism

**Fig. (6):**
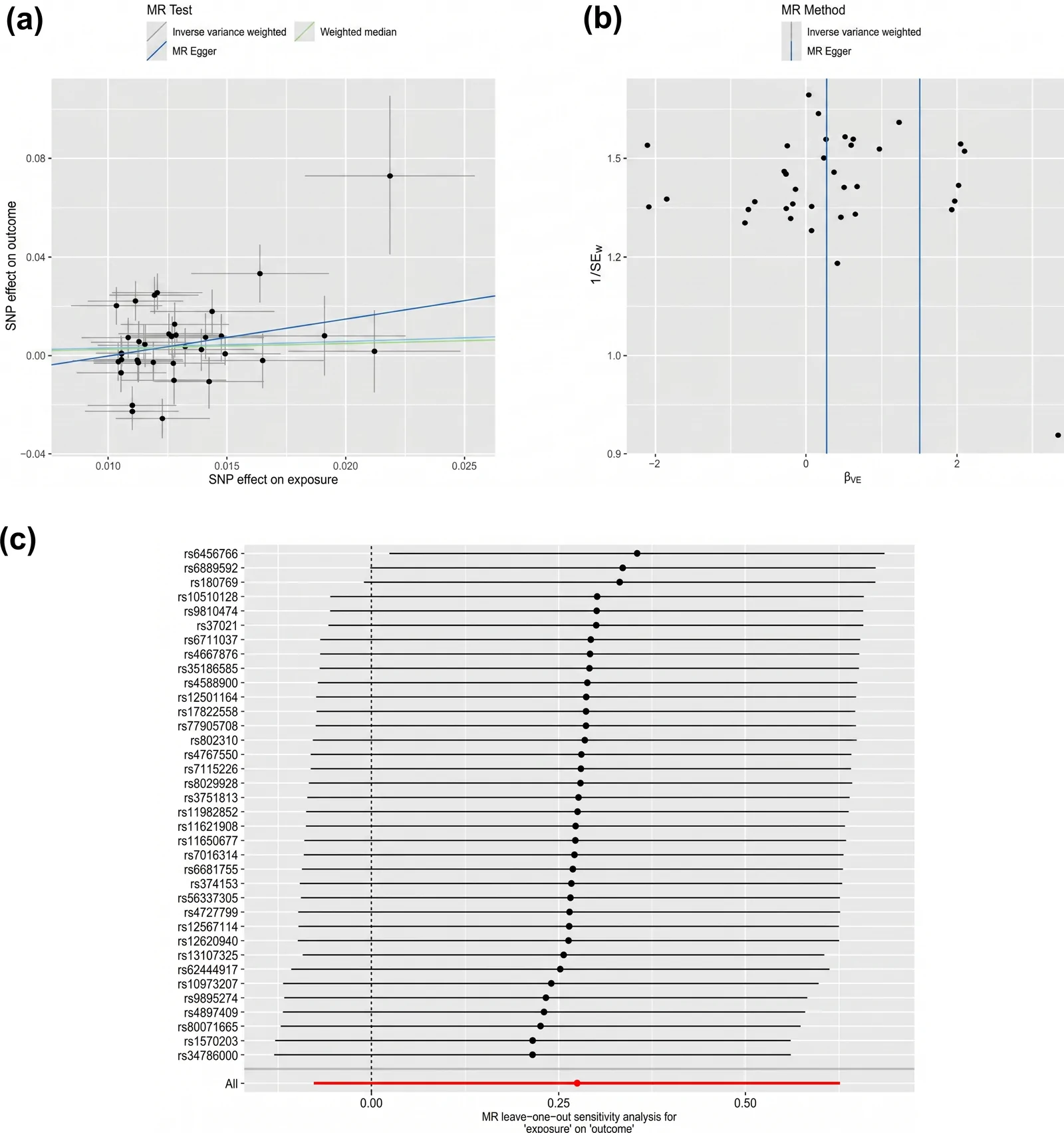
The causal effect of sleep duration on hypothyroidism risk. (**a**) Scatter plot. (**b**) Funnel plot. (**c**) Leave-one-out plot.

The remaining 36 SNPs were incorporated into the ensuing MR analysis. The IVW analysis revealed no significant correlation between sleep duration and hypothyroidism (IVW, OR 1.3163; 95% CI 0.9261-1.8708; *P*=0.1255). Both the MR-Egger and weighted median approaches exhibited no substantial divergence from the IVW intercept. The MR-PRESSO method identified two outliers (rs6456766 and rs6889592), and following their exclusion, the MR analysis revealed a positive association between sleep duration and hypothyroidism (IVW, OR 1.5208; 95% CI 1.1142-2.0758; *P*=0.0082), despite the persistence of significant heterogeneity (*Q*=57.30, *P*=0.0054). The MR-Egger regression revealed no indication of horizontal pleiotropy (*P*=0.4774). The data were substantial, indicating no weak instrument bias (F=34.60), and the leave-one-out analysis verified that the observed correlation was unaffected by any one SNP. The MVMR analysis revealed no correlation between sleep duration and hypothyroidism after controlling for smoking and alcohol consumption (Fig. **6**). 

#### 3.5.3 Nap during the Day and Hypothyroidism

**Fig. (7):**
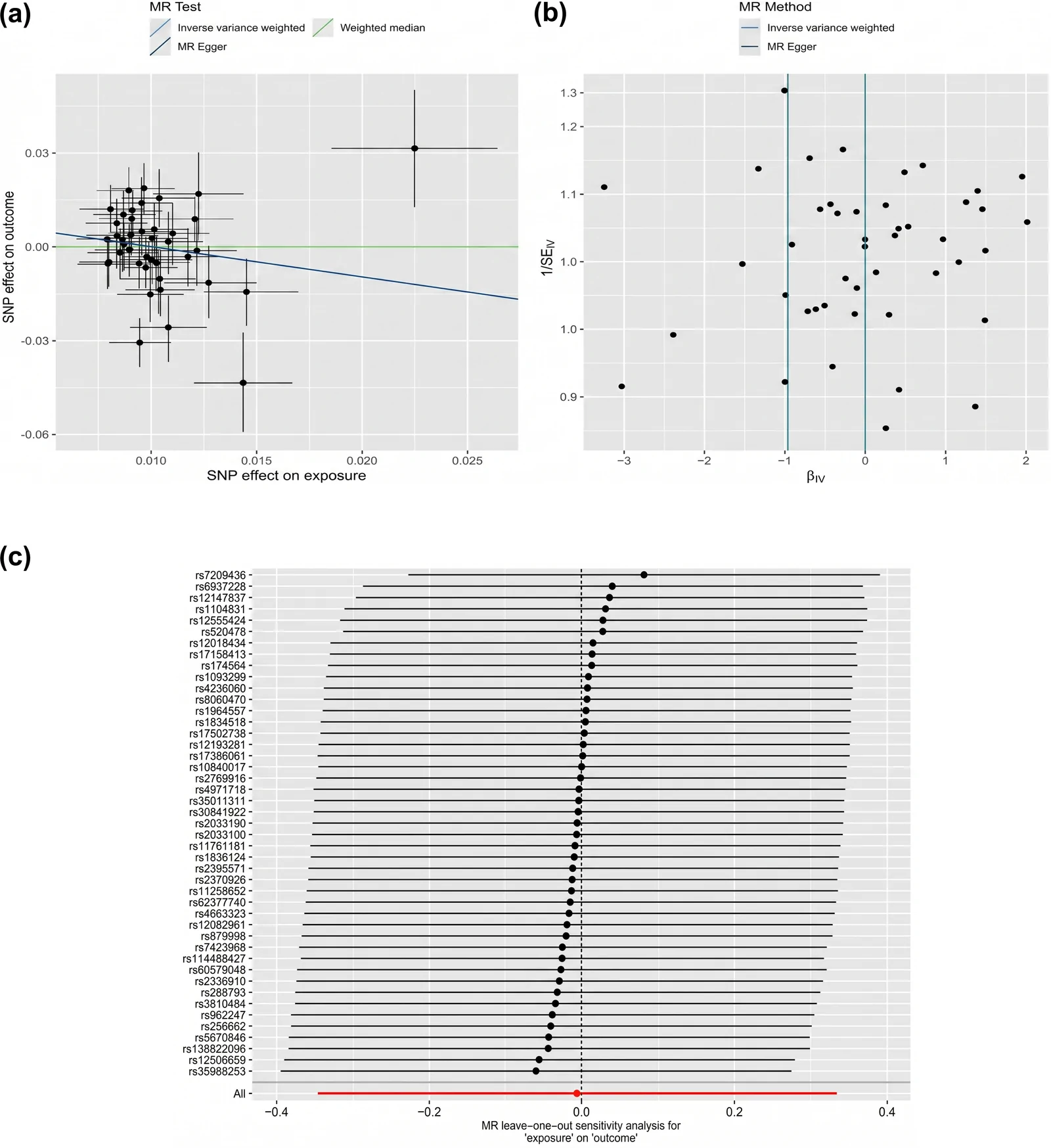
The causal effect of nap during day on hypothyroidism risk. (**a**) Scatter plot. (**b**) Funnel plot. (**c**) Leave-one-out plot.

Following the removal of linkage disequilibrium, 52 SNPs remained. Palindromic and repetitive SNPs not associated with hypothyroidism were then eliminated, resulting in 45 SNPs for the MR analysis. The IVW analysis revealed no significant correlation between naps during the day and hypothyroidism (IVW, OR 0.9939; 95% CI 0.7074-1.3966; *P*=0.9720). Both the MR-Egger and weighted median approaches indicated no substantial divergence from the IVW intercept. The MR-PRESSO technique detected rs7209436 as an anomaly. Upon the exclusion of this SNP, heterogeneity ceased to be significant (*Q*=58.00, *P*=0.0629), and the overarching conclusion remained consistent (IVW, OR 1.0848; 95% CI 0.7964-1.4777, *P*=0.6058). The MR-Egger regression revealed no indication of horizontal pleiotropy (*P*=0.2401). The analysis revealed no evidence of weak instrument bias (F=35.40), and the leave-one-out analysis demonstrated that the results were resilient to genetic variation. The MVMR study revealed no connection between naps during the day and hypothyroidism after controlling for alcohol consumption and smoking (Fig. **7**).

#### 3.5.4 Getting up in the Morning and Hypothyroidism

**Fig. (8):**
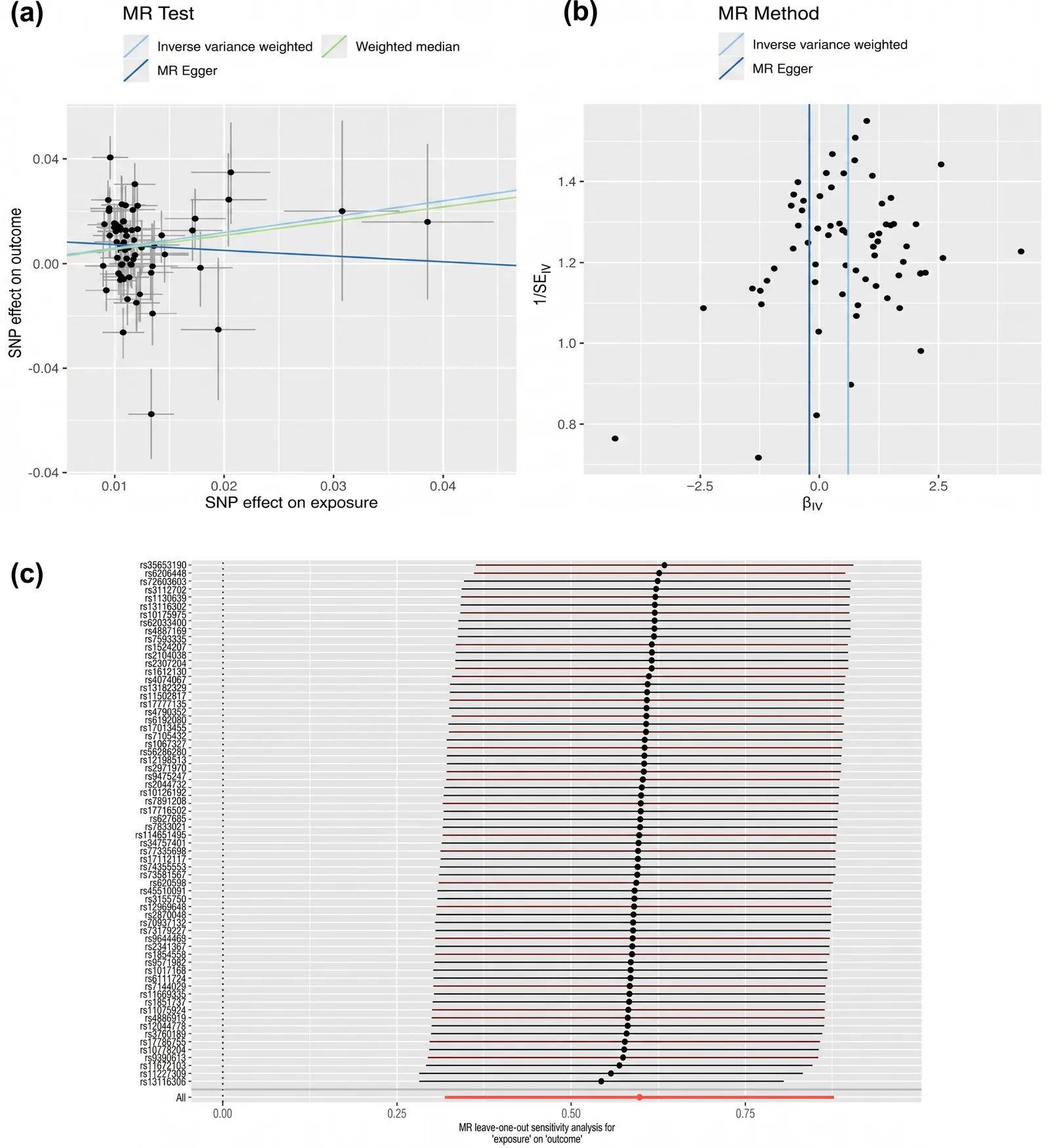
The causal effect of getting up in the morning on hypothyroidism risk. (**a**) Scatter plot. (**b**) Funnel plot. (**c**) Leave-one-out plot.

The IVW analysis demonstrated a positive connection between getting up in the morning and hypothyroidism (IVW, OR 1.8188; 95% CI 1.3759-2.4043; *P*=2.66E-05). The MR- Egger and weighted median approaches exhibited no substantial divergence from the IVW intercept. Following the exclusion of outliers detected by MR-PRESSO, specifically rs13116306, rs35653190, and rs62065448, the correlation continued to be statistically significant (IVW, OR 1.8375; 95% CI 1.4502-2.3284; *P*=4.73E-07), despite the persistence of heterogeneity (*Q*=88.30, *P*=0.0158). The funnel plot exhibited symmetry, and MR-Egger regression revealed no indication of horizontal pleiotropy (*P*=0.4307). The data exhibited little sensitivity to genetic variation and indicated no evidence of weak instrument bias (F=36.27). The MVMR analysis indicated that, after controlling for alcohol intake and smoking, the causative connection persisted in alignment with the earlier analysis (IVW OR 2.7621; 95% CI: 1.6661–4.5791, *P*=8.17E-05) (Fig. **8**).

#### 3.5.5 Chronotype and Hypothyroidism

**Fig. (9):**
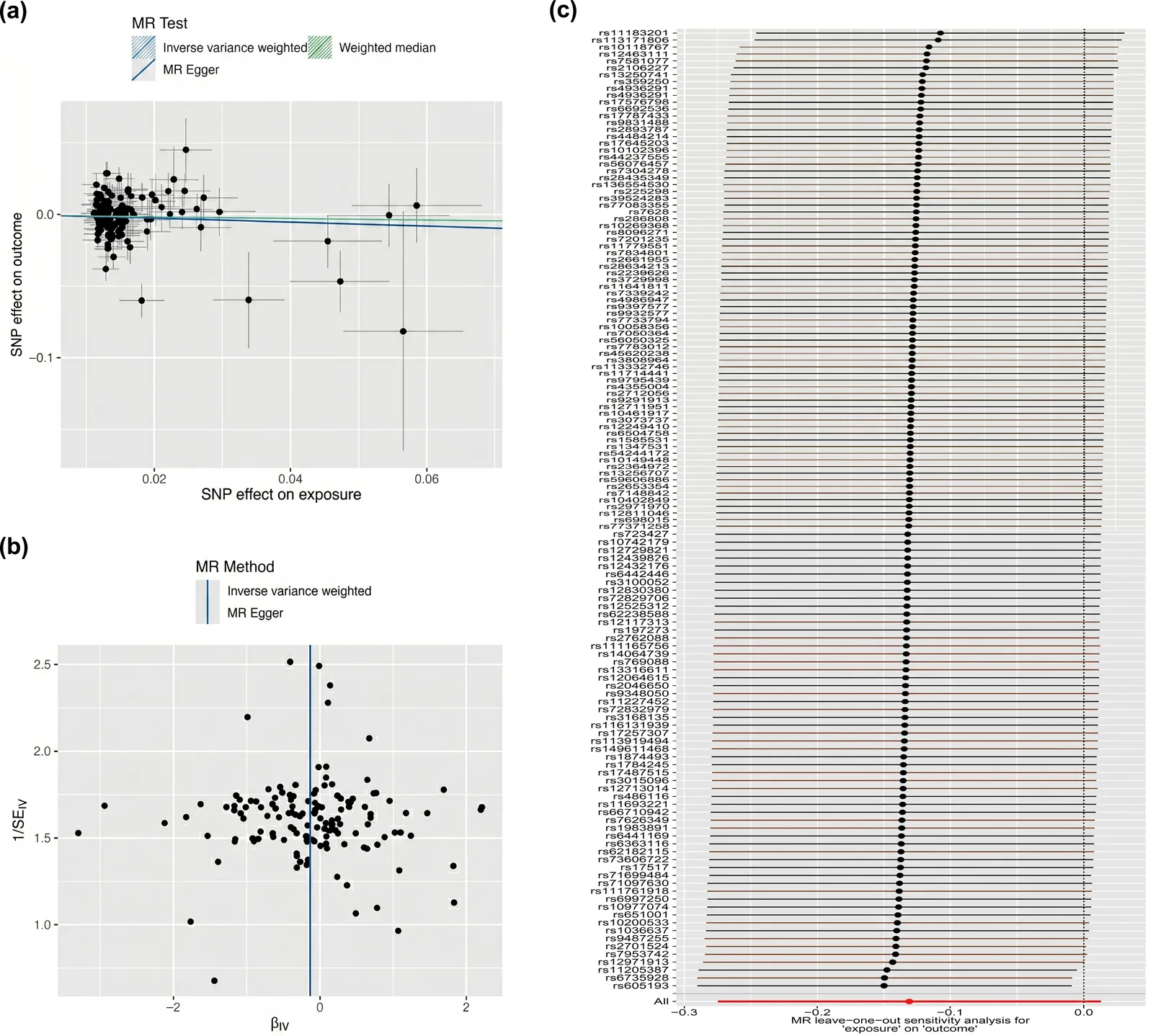
The causal effect of chronotype on hypothyroidism risk. (**a**) Scatter plot. (**b**) Funnel plot. (**c**) Leave-one-out plot.

The IVW analysis revealed no significant correlation between chronotype and hypothyroidism (IVW, OR 0.8773; 95% CI 0.7598-1.0128; *P*=0.0741). Both the MR-Egger regression and weighted median approaches demonstrated no substantial disparity between the MR-Egger intercept and the IVW intercept. The MR-PRESSO approach detected four outliers, but their exclusion did not substantially affect the MR analysis results (IVW, OR 0.8967; 95% CI 0.7799-1.0308; *P*=0.1253), and heterogeneity persisted (*Q*=220.80, *P*=6.61E-07). The funnel plot exhibited symmetry, and MR-Egger regression revealed no indication of horizontal pleiotropy (*P*=0.9863). Furthermore, the results indicated an absence of weak instrumental variable bias (F=36.95), and leave-one-out analysis corroborated no sensitivity to genetic variation. The MVMR study indicated no association between chronotype and hypothyroidism after adjusting for smoking and alcohol consumption (Fig. **9**).

#### 3.5.6 Snoring and Hypothyroidism

The MR study, which included 33 SNPs, revealed no significant correlation between hypothyroidism and snoring using IVW analysis (IVW, OR 1.4703; 95% CI 0.6923-3.1224; *P*=0.3158). Both the MR-Egger and weighted median approaches demonstrated no statistically significant disparity between the intercept and the IVW intercept (*P*=0.6678 and *P*=0.2712). MR-PRESSO revealed rs2648315 as an outlier; however, its exclusion did not substantially affect the results (IVW, OR 1.2004; 95% CI 0.6206-2.3220; *P*=0.5874), and heterogeneity remained (*Q*=67.51, *P*=0.0002). The MR-Egger regression indicated no evidence of horizontal pleiotropy (*P*=0.4170), and the data revealed no indications of mild instrumental variable bias (F=36.38). The leave-one-out study further validated the absence of sensitivity to genetic variation. In the MVMR analysis, following adjustments for alcohol consumption and smoking, the causative relationship between snoring and hypothyroidism intensified (IVW, OR 6.7233; 95% CI: 2.3074-19.5905, *P*=0.0005). The findings are illustrated in Fig. (**10**).

#### 3.5.7 Hypothyroidism and Sleep Traits

The two-sample MR analysis, utilizing 80 SNPs, showed no evidence of weak instrumental variable bias (F=81.28). The IVW analysis revealed no association between sleep characteristics and hypothyroidism (insomnia: OR 0.9951; 95% CI 0.9886-1.0016; *P*=0.1363; sleep duration: OR 0.9976; 95% CI 0.9896-1.0057; *P*=0.5625; nap during day: OR 0.9994; 95% CI 0.9936-1.0052; *P*=0.8444; get up in the morning: OR 1.0018; 95% CI 0.9936-1.0100; *P*=0.6686; chronotype: OR 1.0034; 95% CI 0.9927-1.0143; *P*=0.5347; snoring: OR 1.0014; 95% CI 0.9966-1.0063; *P*=0.5623). The MR-Egger and weighted median approaches revealed no significant disparity between the intercept and the zero intercept of IVW. Heterogeneity was identified by heterogeneity tests, and the results remained unaffected by outliers. The MR-Egger approach indicated an absence of horizontal pleiotropy between the instrumental components and the outcomes. No genetic variant susceptibility was identified by the leave-one-out study. The forest plot illustrating the results of the forward and reverse MR analysis is presented in Fig. (**11**). Table **5** and Supplementary Table **S1** and **2** provide the detailed MR analysis data, whilst Supplementary Figs (**S1-6**) illustrate the funnel plot, scatter plot, and correlation sensitivity analysis about reverse MR analysis.

## 4 DISCUSSION

The thyroid gland operates suboptimally and secretes inadequate levels of thyroid hormone resulting in a hormonal disorder termed hypothyroidism [44]. Various risk factors, including detrimental behaviors, psychological stress, and inconsistent sleeping habits may contribute to hypothyroidism [45]. The significance of sleep in sustaining a healthy lifestyle and its extensive impact on human immunity is increasingly recognized. Reduced sleep duration may facilitate the onset of hypothyroidism by influencing the hypothalamic-pituitary-thyroid axis, which regulates the secretion of TSH, thyroxine (T4), and triiodothyronine (T3) [46-48]. Reduced sleep duration has been associated with modifications in the release of thyroid hormone, growth hormone, pituitary hormone, and melatonin, with increased expression levels of TNF-a, IL-10, and CRP, as well as a disruption in the circadian rhythm phase system [49]. The immunological and sleep-regulating functions of TNF-a, IL-10, IL-1, and IL-6 collaborate with growth hormone, melatonin, and thyroid hormone to modulate the ratio of helper T cell 2 (Th2) to helper T cell 1 (Th1) cytokine activity [50, 51]. They employ a neuroendocrine mechanism to modulate immunological activation. Furthermore, previous research has identified a correlation between hypothyroidism and both excessive daytime sleepiness and the intensity of snoring [52, 53]. The clinical investigations exhibit limitations such as an ambiguous causal association, a limited sample size, and difficulties in controlling for confounding variables.

In this study, we analyzed the effects of several sleep traits on hypothyroidism, including hypothyroidism, including sleep duration, naps during the day, getting up in the morning, insomnia, chronotype, and snoring. The LDSC outcomes indicated a genetic correlation between hypothyroidism and napping during the day. Importantly, these findings do not indicate a definitive causal relationship between sleep deprivation and the onset of hypothyroidism. A shared genetic basis linking the two categories of disease characteristics. Hypothyroidism may arise from sleep disturbances, which can cause irregular circadian rhythms. This represents a potential explanation. The circadian rhythm components TIMELESS, GHRL, PTEN, NR1D1, and ARNTL modulate the immune system and cell cycle through the glucocorticoid signaling route and insulin signaling pathway [54-56].

The findings of the CPASSOC and color analysis show that most of the shared mapping genes between hypothyroidism and sleep traits congregate in the endocrine, brain, and respiratory systems. Given that hypothyroidism is a category of autoimmune illnesses and is intricately linked to multiple organs and tissues, this concentration suggests a significant association. Significant differences in the allele frequencies of HCP5 (*P*=0.0014, OR=1.50) and MAGI3 (*P*=0.0035, OR=1.50) were found in a study that examined thyroid function in individuals grouped by age of origin. The level of expression will affect TPOAb levels, which will subsequently impact the initiation and progression of the disease [57, 58]. Another cross-sectional clinical investigation found that the polymorphisms HLA-DRB1 and HLA-DQB1 were strongly associated with thyroid autoimmunity [59-61]. HLA-DRB1 and HLA-DQB1 significantly influence cell migration, proliferation, and death by modulating the ERK1/2 signaling pathway and the activities of PI3K, Akt, and cyclin proteins, which are critical factors in several disease processes [62, 63]. Mutations in SKIV2L and PRRC2A have been linked to autoimmune disorders in prior research, particularly with the clinical symptoms of insomnia and hypothyroidism [64, 65]. Despite the paucity of research on BX296568.1, AL731683.1, and BX927168.3.1, their established functions in hypothyroidism and sleep traits demand additional experimental verification.

According to an MR study, the risk of hypothyroidism is heightened by late-night activities and early awakenings. Sleep deprivation adversely affects health. Individuals with hypothyroidism may encounter sleep disturbances, particularly abbreviated sleep durations [66, 67]. Subpar sleep quality and insufficient sleep duration may be contributing factors or causes of hypothyroidism [68, 69]. Individuals with elevated TSH levels demonstrated significantly superior scores on the Pittsburgh Sleep Quality Index (PSQI) for subjective sleep quality, sleep latency, and sleep duration in both cross-sectional and longitudinal studies. A strong positive connection was seen between shorter sleep duration and elevated TSH levels (OR=2.396, *P*=0.001) in multivariate logistic regression models [70]. A separate study indicated that night workers were 1.399 times more predisposed to developing hypothyroidism (95% CI: 1.050-1.863, *P*=0.022) and exhibited TSH levels 0.303 mIU/L elevated compared to the general population (95% CI: 0.087-0.519 mIU/L, *P*=0.006) [71]. The rationale for these linkages remains uncertain. Hypothyroidism may arise from sleep disturbances, which can cause irregular circadian rhythms. This represents a potential explanation. Blood transcriptome sequencing indicates that immune dysfunction significantly contributes to the onset of hypothyroidism. These findings align with our own and suggest that hypothyroidism is negatively influenced by inconsistent sleep length. Furthermore, MR analysis revealed an association between hypothyroidism and challenges in morning awakening, perhaps providing a focus for forthcoming mechanistic investigations. Clinical studies indicate that individuals with sleep disorders, such as insomnia and reduced nocturnal sleep, often struggle to awaken in the morning [72]. Insufficient study has been conducted on the challenges of waking in the morning, and there is a scarcity of relevant foundational experimental studies and pharmacological advancements, which may represent significant avenues for additional exploration.

Clinical study results have not consistently aligned [73, 74]. Akatsu *et al.* found no significant differences in sleep quality, including duration, efficiency, latency, and tiredness, when comparing individuals with subclinical hypothyroidism to those without it [75]. A reverse magnetic resonance scan revealed that hypothyroidism did not increase the risk of insomnia, prolonged sleep duration, snoring, or daytime napping. Hypothyroidism is not a risk factor for sleep disorders; nevertheless, symptoms of thyroid hormone deficiency, including anxiety, chills, and musculoskeletal pain, may aggravate the onset of sleep problems.

This study identifies potential common genes, explores the underlying mechanisms of action of these genes, and emphasizes the importance of adopting improved sleeping habits. Subsequent clinical research in this domain may expand upon these results. The study has several limitations. The applicability of the MR analysis to other populations is uncertain and requires further investigation, as it was conducted on a large cohort of European origin. Secondly, ascertaining the precise extent of subject overlap remains challenging, especially when employing robust techniques to mitigate sample overlap bias. Ultimately, there exists a possibility that confounding variables may result in exposure misclassification due to the self-reported nature of all sleep metrics. Consequently, additional confirmation through fundamental research or clinical investigations is necessary.

## CONCLUSION

Our study has presented compelling evidence of genetic associations, and identified shared loci, thereby exploring network associations between multiple sleep traits and hypothyroidism. While other sleep traits do not prevent or adversely influence the development of hypothyroidism, these results imply that attention to sleep health is still required to lower the risk of hypothyroidism. These findings enhance our understanding of observational associations and provide robust support for causal inferences.

## Figures and Tables

**Fig. (1):**
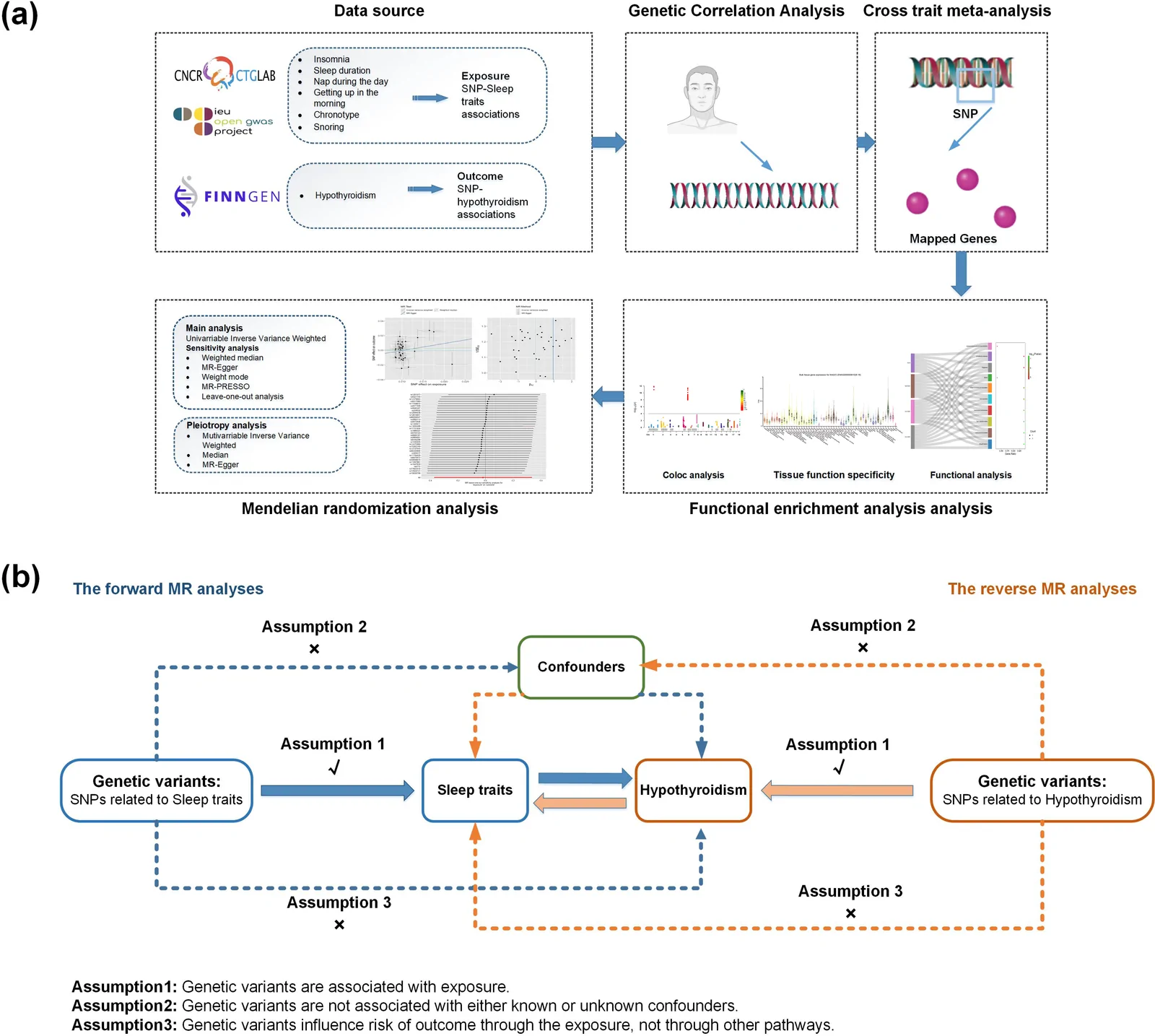
Overall study design. (**a**) Flowchart of this study. (**b**) The principles and main design of Mendelian Randomization study.

**Fig (2):**
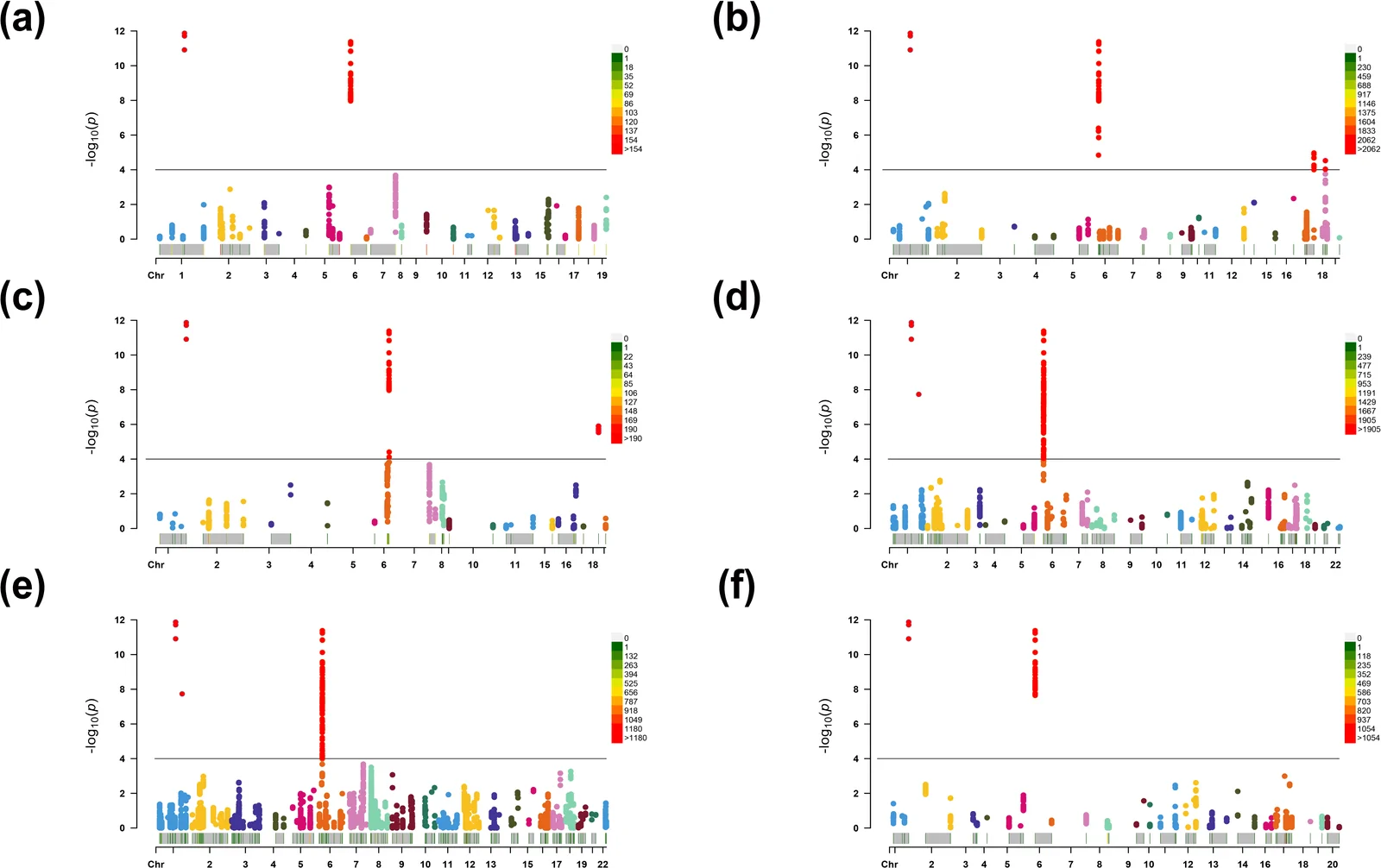
The manhattan plots of the shared genes based on results of CPASSOC between sleep traits and hypothyroidism. (**a**) insomnia. (**b**) sleep duration. (**c**) nap during the day. (**d**) getting up in the morning. (**e**) chronotype. (**f**) snoring.

**Fig. (10):**
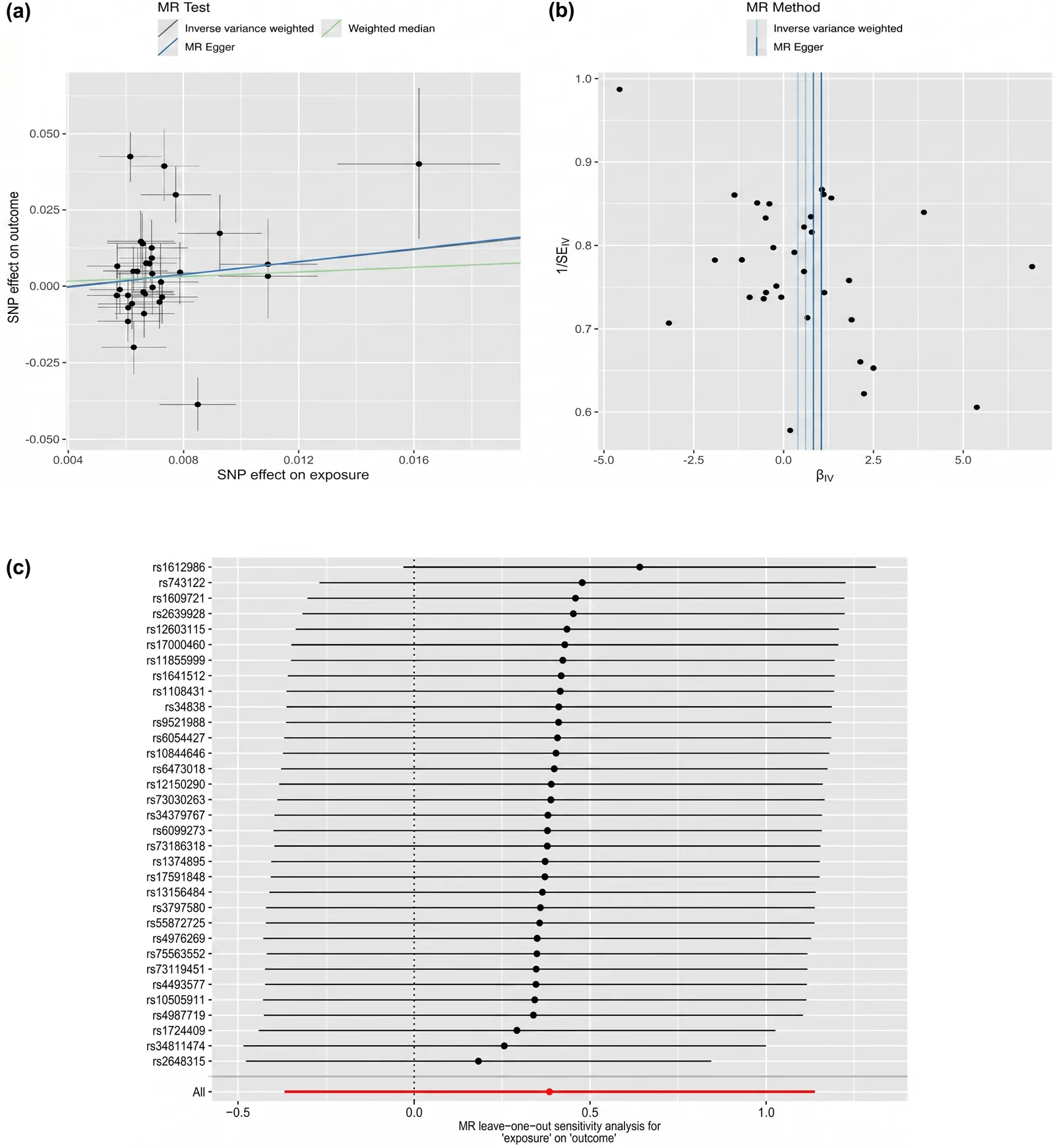
The causal effect of snoring on hypothyroidism risk. (**a**) Scatter plot. (**b**) Funnel plot. (**c**) Leave-one-out plot.

**Fig. (11):**
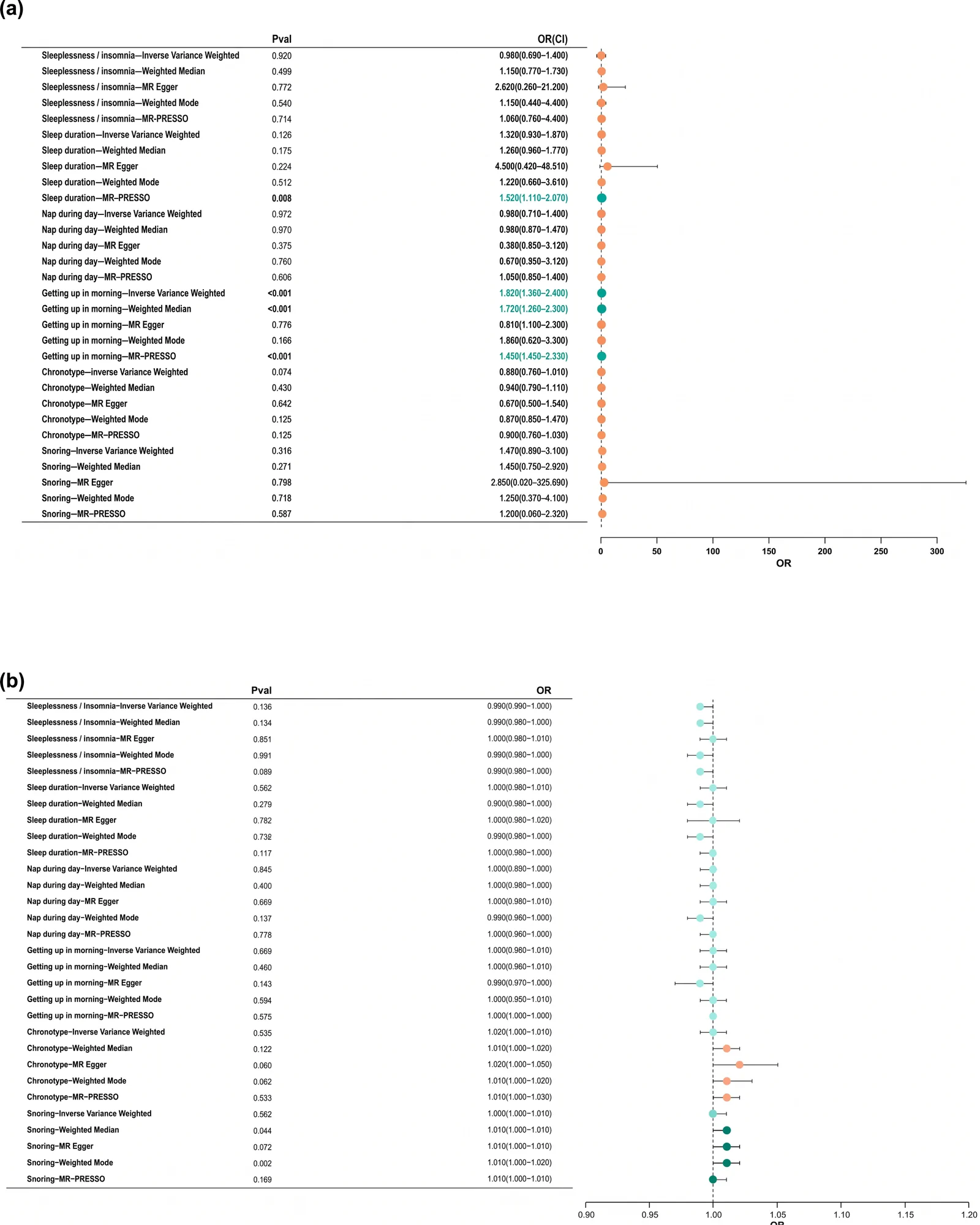
The forest plots for forward (**a**) and reverse (**b**) MR analysis.

**Table 1:** The GWAS data source of sleep traits and hypothyroidism.

**Exposure/Outcome**	**GWAS ID**	**Sample Size**	**Number of SNPs**	**Population**	**Consortium**	**Source**
Sleeplessness / insomnia	ukb-b-3957	462,341	9,851,867	European	MRC-IEU	https://gwas.mrcieu.ac.uk/
Sleep duration	ukb-a-9	335,410	10,894,596	European	Neale Lab	https://gwas.mrcieu.ac.uk/
Nap during day	ukb-a-12	337,074	10,894,596	European	Neale Lab	https://gwas.mrcieu.ac.uk/
Getting up in morning	ukb-b-2772	461,658	9,851,867	European	MRC-IEU	https://gwas.mrcieu.ac.uk/
Chronotype	ukb-b-4956	413,343	9,851,867	European	MRC-IEU	https://gwas.mrcieu.ac.uk/
Snoring	ukb-b-17400	430,438	9,851,867	European	MRC-IEU	https://gwas.mrcieu.ac.uk/
Alcoholic drinks per week	ieu-b-73	335,394	11,887,865	European	GWAS and Sequencing Consortium of Alcohol and Nicotine use	https://gwas.mrcieu.ac.uk/
Smoking initiation	ieu-b-4877	607,291	11,802,365	European	GSCAN	https://gwas.mrcieu.ac.uk/
Hypothyroidism	finn-b-E4_HYTHYNAS	334,316	16,380,461	European	NA	https://www.finngen.fi/en

**Table 2:** Genetic correlation between sleep traits and hypothyroidism.

**Sleep Traits**	**Genetic Correlations (SE)**	***P*-value**
Sleeplessness / insomnia	0.0238 (0.0304)	0.4331
Sleep duration	-0.0245 (0.0328)	0.4545
Nap during day	-0.0982 (0.0288)	0.0007
Getting up in morning	-0.101 (0.0262)	0.0001
Chronotype	0.0456 (0.0382)	0.2322
Snoring	-0.0281 (0.028)	0.3154

**Table 3:** Genome-wide significant loci in genome-wide cross-trait analysis methods.

**GWAS ID**	**SNP**	**Gene**	**Chrom**	**REF**	**ALT**	**ES**	**SE**	***P*-value**	**p_Shet**	**p_Shom**
ukb-b-3957	rs1230666	MAGI3	1	A	G	-0.005	0.002	1.23E-11	5.63E-11	5.57E-08
	rs28533694	-	6	C	T	-0.001	0.002	7.34E-10	3.36E-09	1.49E-04
	rs9270493	HLA-DRB1, BX296568.1	6	T	C	-0.002	0.002	3.27E-09	1.49E-08	3.38E-02
	rs9273363	HLA-DQB1, HLA-DQA1, AL731683.1, BX927168.3, BX927168.1, AL935026.1	6	C	A	0.000	0.002	3.41E-10	1.56E-09	1.09E-03
ukb-a-9	rs1230666	MAGI3	1	A	G	-0.003	0.003	1.23E-11	3.39E-11	2.04E-07
	rs28533694	-	6	C	T	-0.003	0.002	7.34E-10	2.22E-09	4.89E-07
	rs604760	CELF4	18	A	G	0.011	0.003	1.26E-06	1.07E-08	6.39E-01
	rs9270493	HLA-DRB1, BX296568.1	6	T	C	0.003	0.002	3.27E-09	1.01E-08	2.36E-06
	rs9273363	HLA-DQB1, HLA-DQA1, AL731683.1, BX927168.3, BX927168.1, AL935026.1	6	C	A	0.001	0.002	3.41E-10	1.01E-09	9.61E-06
ukb-a-12	rs1230666	MAGI3	1	A	G	0.006	0.002	1.23E-11	3.12E-11	2.52E-02
	rs204991	NOTCH4, GPSM3, PBX2	6	T	C	0.007	0.002	1.38E-06	3.25E-09	1.13E-09
	rs28533694	-	6	C	T	0.000	0.002	7.34E-10	2.09E-09	3.59E-05
	rs2854002	HLA-B	6	A	G	0.006	0.002	5.72E-07	2.78E-08	9.22E-09
	rs9270493	HLA-DRB1, BX296568.1	6	T	C	0.001	0.002	3.27E-09	9.61E-09	8.36E-06
	rs9273363	HLA-DQB1, HLA-DQA1, AL731683.1, BX927168.3, BX927168.1, AL935026.1	6	C	A	0.001	0.002	3.41E-10	9.49E-10	1.34E-06
ukb-b-2772	rs1230666	MAGI3	1	A	G	0.000	0.002	1.23E-11	2.33E-11	2.48E-04
	rs3130477	HCP5	6	T	C	0.008	0.002	4.28E-07	4.88E-08	1.88E-08
	rs389884	DXO, STK19, SKIV2L	6	A	G	0.011	0.002	5.10E-07	1.08E-10	5.32E-11
	rs9270493	HLA-DRB1, BX296568.1	6	T	C	0.007	0.002	3.27E-09	1.86E-09	8.11E-10
	rs9270664	HLA-DRB1	6	G	A	0.002	0.002	1.72E-08	4.45E-08	6.24E-05
	rs9273873	HLA-DQB1, HLA-DQA1, AL731683.1, BX927168.3, BX927168.1, AL935026.1	6	T	C	0.007	0.002	3.27E-07	3.37E-09	1.44E-09
ukb-b-4956	rs1230666	MAGI3	1	A	G	0.006	0.003	1.23E-11	2.24E-11	2.88E-02
	rs1281937	HLA-DRB1	6	T	C	-0.005	0.002	1.09E-08	2.67E-08	1.16E-01
	rs3132450	PRRC2A, BX511262.2	6	A	G	-0.011	0.003	1.52E-07	2.34E-09	9.64E-01
	rs3132941	PPT2, EGFL8, AGPAT1	6	G	C	-0.011	0.003	1.09E-07	4.60E-09	8.97E-01
	rs35242582	HLA-DQA1	6	A	G	0.009	0.002	1.64E-06	3.41E-08	8.84E-01
	rs9270664	HLA-DRB1, BX296568.1	6	G	A	-0.004	0.002	1.72E-08	4.32E-08	1.00E-01
ukb-b-17400	rs1230666	MAGI3	1	A	G	0.003	0.001	1.23E-11	1.46E-11	2.29E-02
	rs9270493	HLA-DRB1, BX296568.1	6	T	C	0.000	0.001	3.27E-09	5.63E-09	3.92E-04
	rs9270664	HLA-DRB1, BX296568.1	6	G	A	-0.001	0.001	1.72E-08	3.29E-08	3.50E-02

**Table 4:** Results from colocalization analysis for each pleiotropic locus identified from CPASSOC.

**SNP**	**Position**	**Chromosome**	**A1**	**A2**	**Beta**	**SE**	***P*-value**	**MAF**	**SNP.PP.H4**
rs2476601	114080958	1	A	G	-0.0357	0.0136	8.45E-03	0.1002	0.9980
rs1049225	32627747	6	A	G	0.7425	0.0205	2.02E-287	0.2480	0.9999
rs9271638	32592142	6	C	T	0.4096	0.0110	1.07E-301	0.3752	0.9997
rs9273330	32623300	6	C	T	0.5343	0.0143	1.78E-304	0.4444	0.9999
rs17843604	32620283	6	T	C	-0.0679	0.0146	3.19E-06	0.5170	0.9866
rs1800628	31546850	6	A	G	-0.2462	0.0221	9.96E-29	0.1043	0.7498
rs6472	32007849	6	C	G	0.6666	0.0183	4.46E-292	0.1167	0.9999
rs9270911	32572202	6	C	T	-0.0224	0.0165	1.74E-01	0.4651	0.9999
rs9272320	32604124	6	G	A	0.0694	0.0142	1.00E-06	0.4840	0.8687

**Table 5:** MVMR for the effect of sleep traits on hypothyroidism.

**Adjustment**	**Exposure**	**Outcome**	**Nsnp**	**Method**	**OR (95%CI)**	**β**	**SE**	***P*-value**	***P*(Heterogeneity)**
Alcoholic drinks per week Smoking initiation	Sleeplessness / insomnia	Hypothyroidism	13	Inverse Variance Weighted	1.10(0.68,1.77)	0.096	0.243	0.692	0.4568
				Median	-	0.280	0.373	0.453	-
				MR Egger		-0.108	0.310	0.728	
	Sleep duration		21	Inverse Variance Weighted	0.73(0.43,1.24)	-0.313	0.268	0.243	0.0035
				Median	-	-0.276	0.293	0.345	-
				MR Egger		-0.215	0.296	0.467	
	Nap during day		23	Inverse Variance Weighted	0.78(0.53,1.15)	-0.245	0.198	0.216	0.3722
				Median	-	0.034	0.277	0.902	-
				MR Egger		-0.242	0.204	0.236	
	Getting up in morning		31	Inverse Variance Weighted	2.76(1.67,4.58)	1.016	0.258	***0.000***	0.0000
				Median	-	1.439	0.295	0.000	-
				MR Egger		0.994	0.261	0.000	
	Chronotype		53	Inverse Variance Weighted	0.92(0.73,1.16)	-0.083	0.117	0.474	0.0000
				Median	-	0.028	0.135	0.835	-
				MR Egger		-0.091	0.117	0.435	
	Snoring		15	Inverse Variance Weighted	6.72(2.31,19.59)	1.906	0.546	***0.001***	0.0185
				Median	-	1.733	0.744	0.020	-
				MR Egger		1.949	0.568	0.001	

**Abbreviations:** Nsnp, Number of used SNPs; OR, odd ratio; CI, condifence interval; IVW, inverse variance weighted; MR, Mendelian randomization; β, beta; SE, standard error.

## Data Availability

The summary GWAS statistics can be downloaded from the websites: IEU open gwas project (https://gwas.mrcieu.ac.uk/), UK Biobank (http://www.nealelab.is/uk-biobank), and FINNGEN (https://www.finngen.fi/fi). All the analyses used in this study were performed in R packages. The code is available at https://mrcieu.github.io/TwoSampleMR/ and https://github.com/n-mounier/MRlap.

## LIST OF ABBREVIATIONS

TPOAb: Thyroid Peroxidase Antibody
GWAS: Genome-wide Association Studies
LDSC: linkage Disequilibrium Score Regression
LD: Linkage Disequilibrium
SNP: Single Nucleotide Polymorphism
SHet: Sample Heterogeneity
VEP: Variant Effect Predictor
GTEx: Genotype Tissue Expression Project
IVW: Inverse-Variance Weighted
