# Supplementary material for: Shared Genetic Basis, Biological Function and Causal Relationship Between Sleep Traits and Hypothyroidism: Evidence from a Comprehensive Genetic Analysis
Source: Endocr Metab Immune Disord Drug Targets. 2025 Apr 3;26:E18715303348704. doi: 10.2174/0118715303348704250227014802 (PMC13523181; doi:10.2174/0118715303348704250227014802)
Supplement: Supplementary file 1 [file EMIDDT-26-E18715303348704_SD1.pdf]

# Supplementary Material

## Shared Genetic Basis, Biological Function and Causal Relationship Between Sleep Traits and Hypothyroidism: Evidence from a Comprehensive Genetic Analysis

Yinli Shi[1,#], Ming Guo[2,#], Yuedan Wang[3,#], Guoduan Zeng[4], Wenting Li[5], Mianhua Wu[5] and Bo Li[5,*]

*[1]Institute Of Basic Research In Clinical Medicine, China Academy of Chinese Medical Sciences, Beijing, 100700, China; [2]Zhongda Hospital Southeast University, Southeast University, Nanjing, 210023, China; [3]Institute of Digestive Diseases, Xiyuan Hospital of China Academy of Chinese Medical Sciences, Beijing, 100091, China; [4]Jinjiang Hospital of Traditional Chinese Medicine, Fujian University of Chinese Medicine, Fujian, 362211, China; [5]The First Clinical Medical College, Nanjing University of Chinese Medicine, Nanjing, 210023, China*

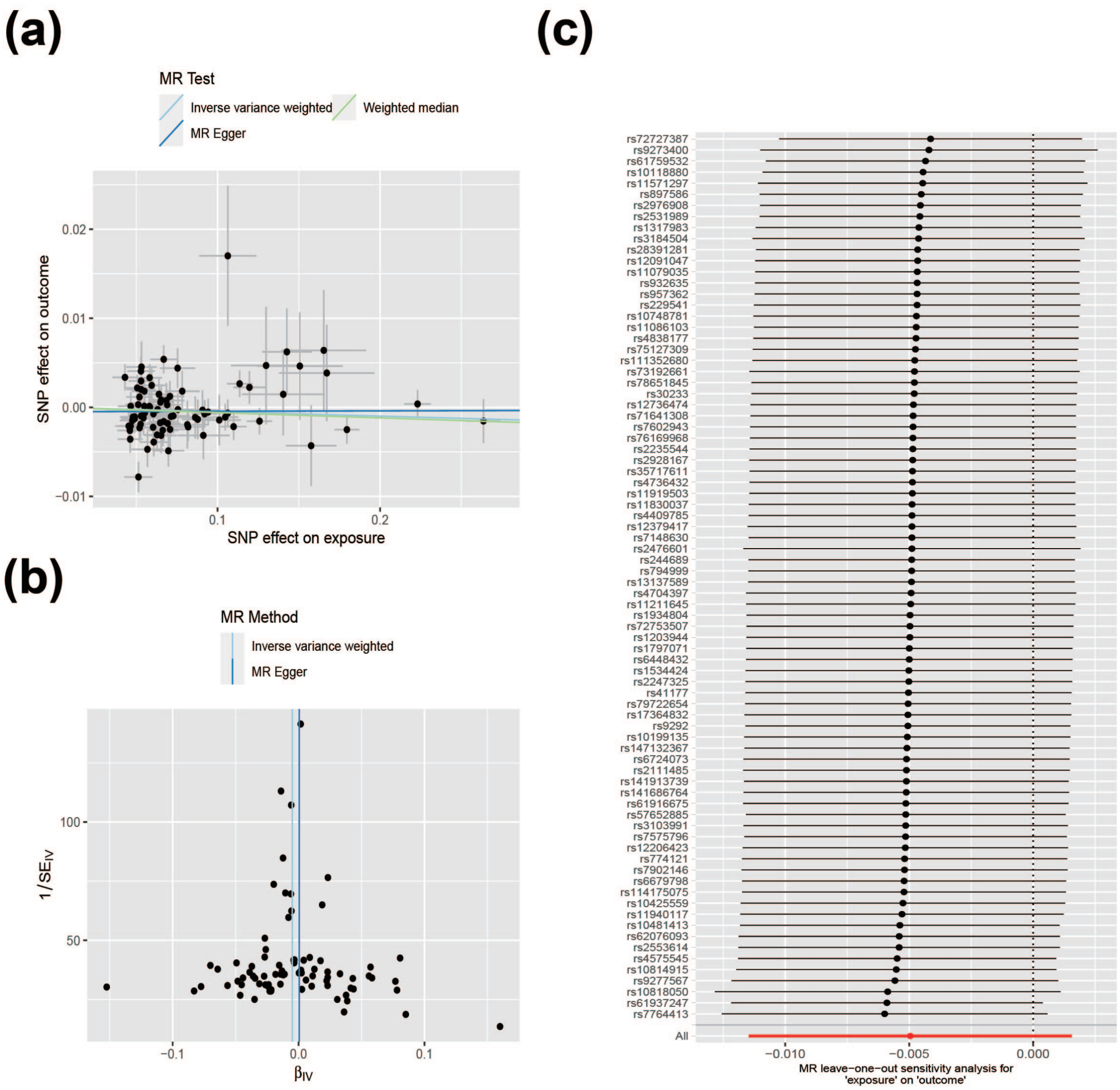


**Supplementary Fig. (1)**

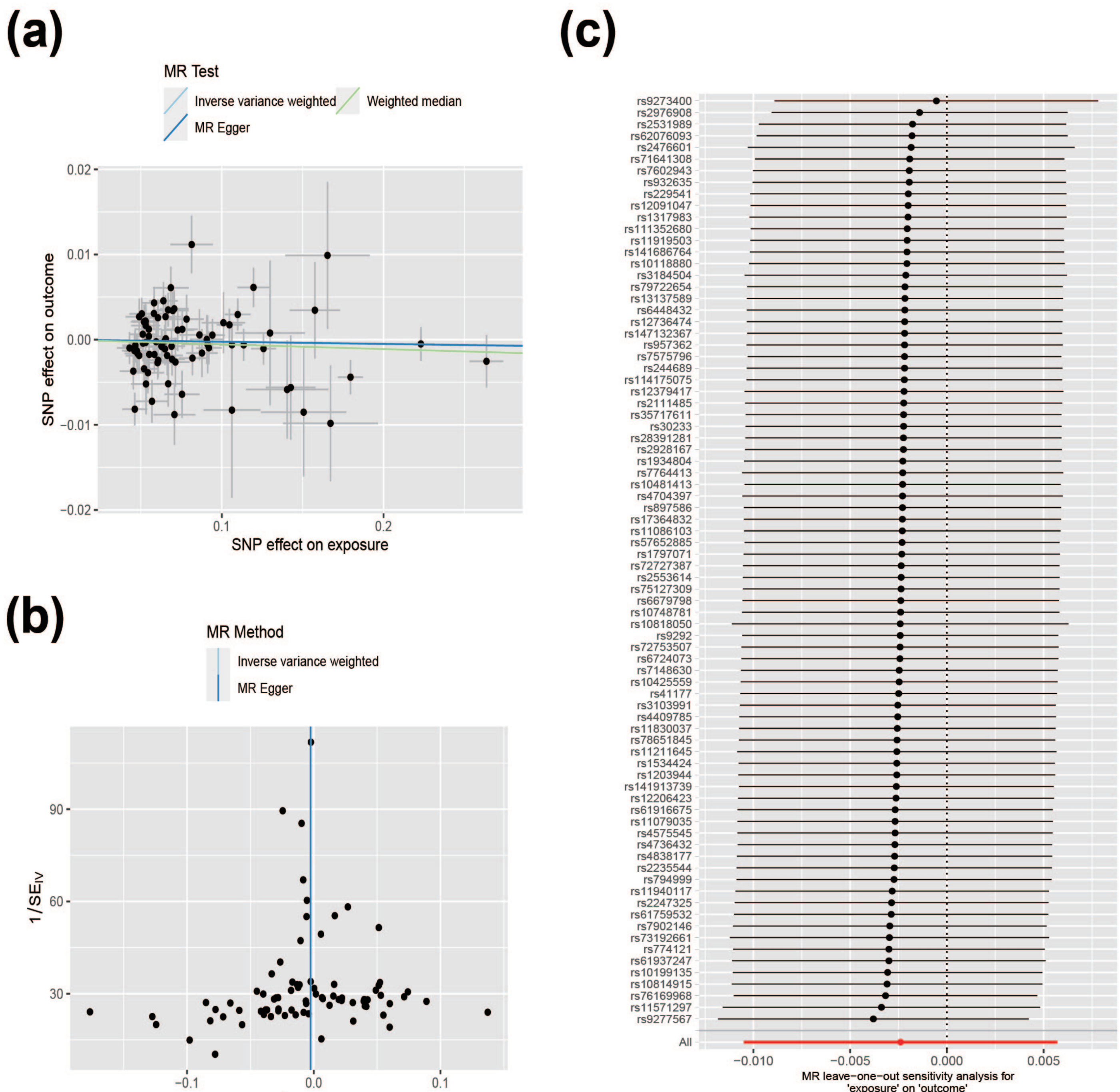


**Supplementary Fig. (2)**

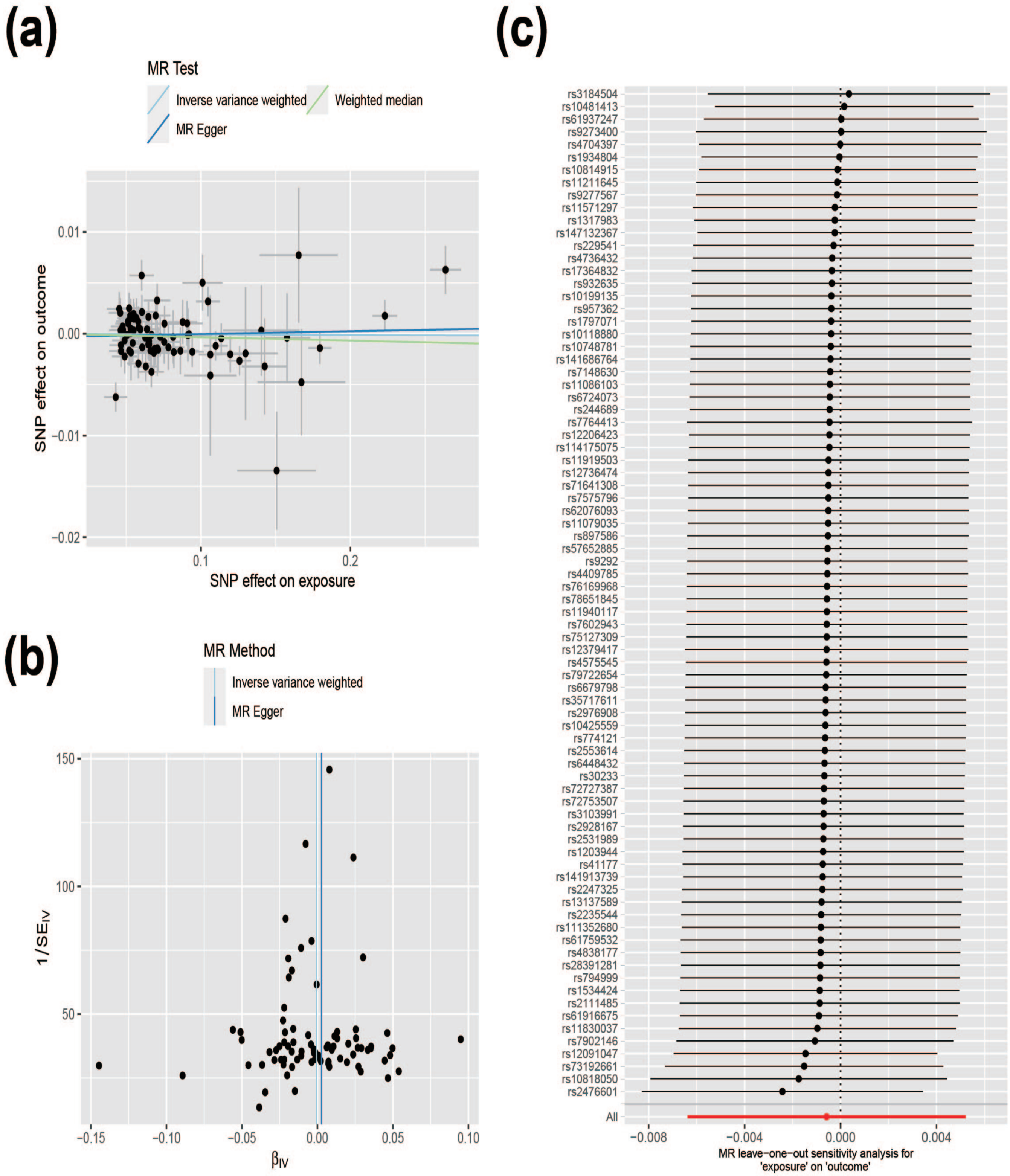


**Supplementary Fig. (3)**

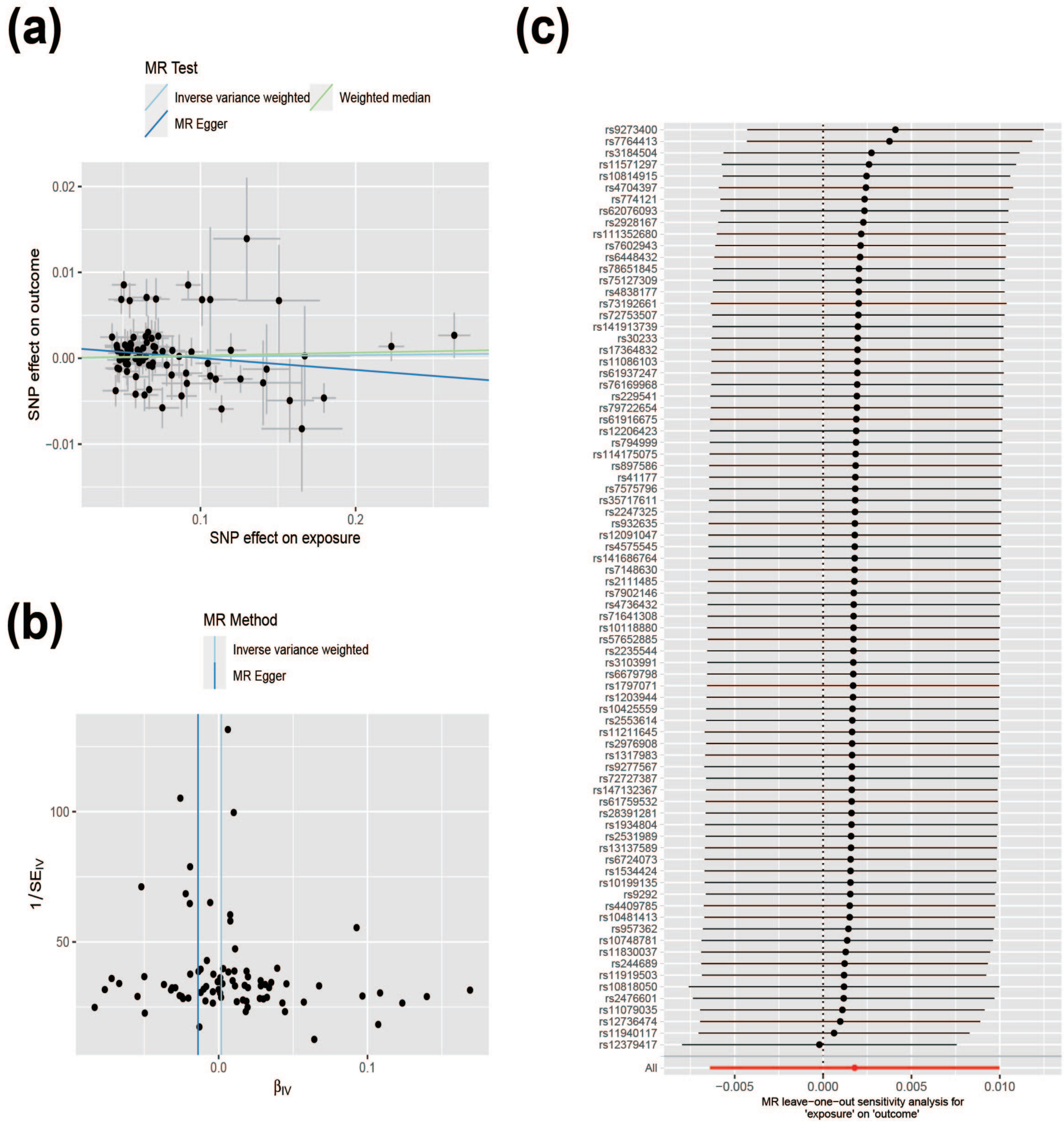


**Supplementary Fig. (4)**

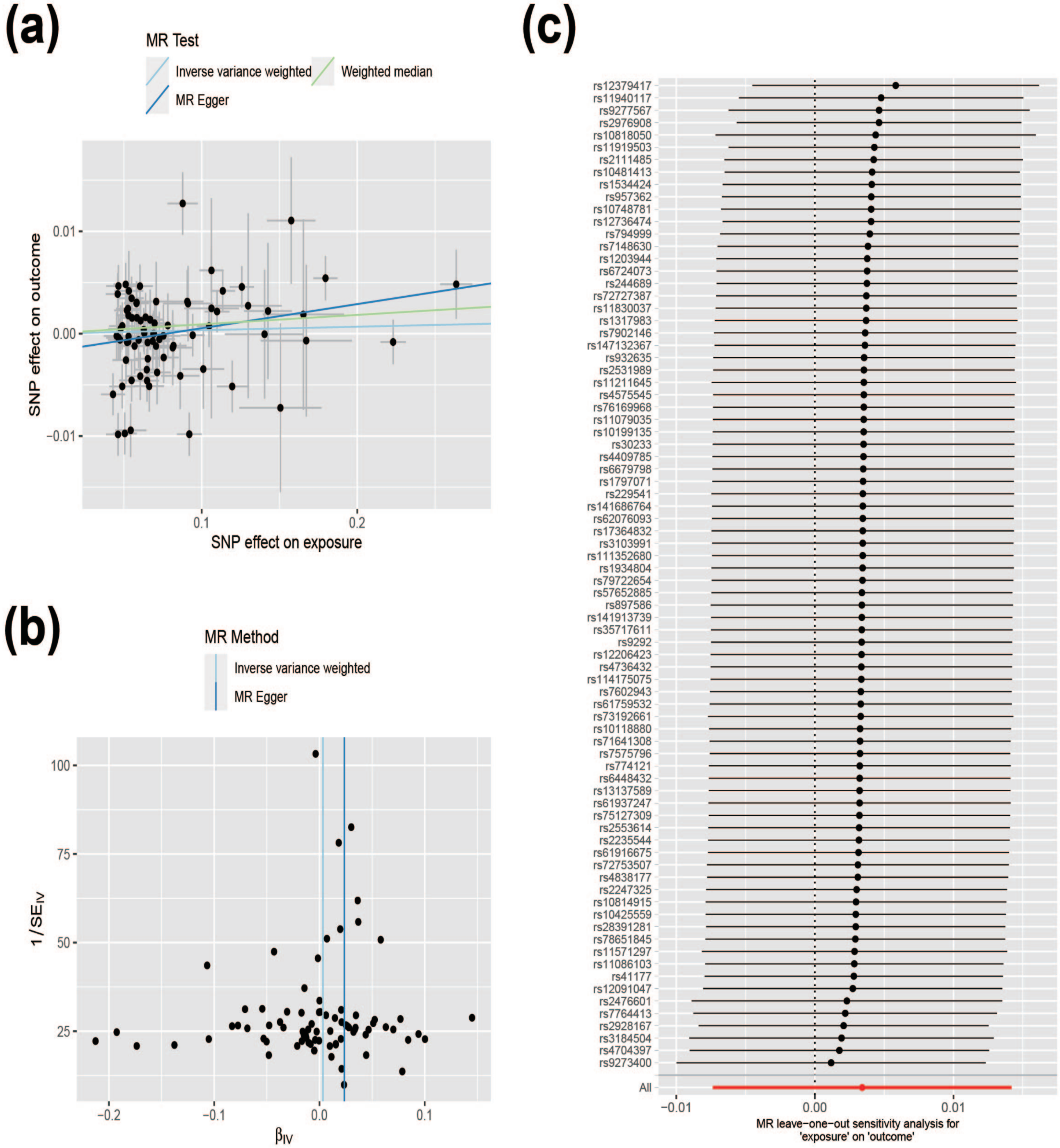


**Supplementary Fig. (5)**

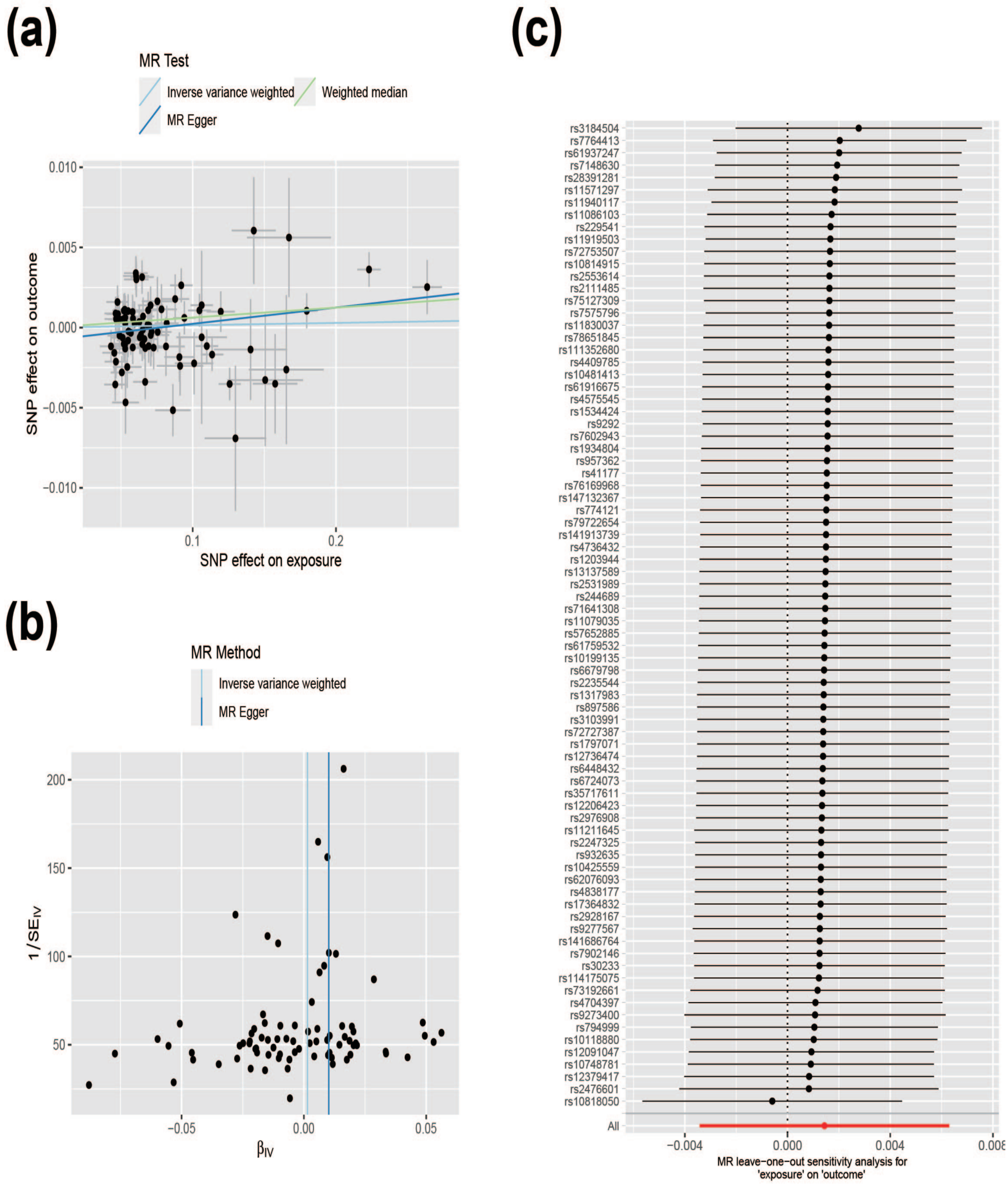


**Supplementary Fig. (6)**
